# The Influence of Anthropomorphic Cues on Patients’ Perceived Anthropomorphism, Social Presence, Trust Building, and Acceptance of Health Care Conversational Agents: Within-Subject Web-Based Experiment

**DOI:** 10.2196/44479

**Published:** 2023-08-10

**Authors:** Qingchuan Li, Yan Luximon, Jiaxin Zhang

**Affiliations:** 1 School of Humanities and Social Sciences Harbin Institute of Technology Shenzhen China; 2 School of Design The Hong Kong Polytechnic University Hong Kong China; 3 School of System Design and Intelligent Manufacturing Southern University of Science and Technology Shenzhen China

**Keywords:** anthropomorphic cues, intelligent guidance conversational agents, social presence, trust, technology acceptance, mindful and mindless anthropomorphism

## Abstract

**Background:**

The last decade has witnessed the rapid development of health care conversational agents (CAs); however, there are still great challenges in making health care CAs trustworthy and acceptable to patients.

**Objective:**

Focusing on intelligent guidance CAs, a type of health care CA for web-based patient triage, this study aims to investigate how anthropomorphic cues influence patients’ perceived anthropomorphism and social presence of such CAs and evaluate how these perceptions facilitate their trust-building process and acceptance behavior.

**Methods:**

To test the research hypotheses, the video vignette methodology was used to evaluate patients’ perceptions and acceptance of various intelligent guidance CAs. The anthropomorphic cues of CAs were manipulated in a 3×2 within-subject factorial experiment with 103 participants, with the factors of agent appearance (high, medium, and low anthropomorphic levels) and verbal cues (humanlike and machine-like verbal cues) as the within-subject variables.

**Results:**

The 2-way repeated measures ANOVA analysis indicated that the higher anthropomorphic level of agent appearance significantly increased mindful anthropomorphism (high level>medium level: 4.57 vs 4.27; *P*=.01; high level>low level: 4.57 vs 4.04; *P*<.001; medium level>low level: 4.27 vs 4.04; *P*=.04), mindless anthropomorphism (high level>medium level: 5.39 vs 5.01; *P*<.001; high level>low level: 5.39 vs 4.85; *P*<.001), and social presence (high level>medium level: 5.19 vs 4.83; *P*<.001; high level>low level: 5.19 vs 4.72; *P*<.001), and the higher anthropomorphic level of verbal cues significantly increased mindful anthropomorphism (4.83 vs 3.76; *P*<.001), mindless anthropomorphism (5.60 vs 4.57; *P*<.001), and social presence (5.41 vs 4.41; *P*<.001). Meanwhile, a significant interaction between agent appearance and verbal cues (.004) was revealed. Second, the partial least squares results indicated that privacy concerns were negatively influenced by social presence (β=−.375; *t*_312_=4.494) and mindful anthropomorphism (β=−.112; *t*_312_=1.970). Privacy concerns (β=−.273; *t*_312_=9.558), social presence (β=.265; *t*_312_=4.314), and mindless anthropomorphism (β=.405; *t*_312_=7.145) predicted the trust in CAs, which further promoted the intention to disclose information (β=.675; *t*_312_=21.163), the intention to continuously use CAs (β=.190; *t*_312_=4.874), and satisfaction (β=.818; *t*_312_=46.783).

**Conclusions:**

The findings show that a high anthropomorphic level of agent appearance and verbal cues could improve the perceptions of mindful anthropomorphism and mindless anthropomorphism as well as social presence. Furthermore, mindless anthropomorphism and social presence significantly promoted patients’ trust in CAs, and mindful anthropomorphism and social presence decreased privacy concerns. It is also worth noting that trust was an important antecedent and determinant of patients’ acceptance of CAs, including their satisfaction, intention to disclose information, and intention to continuously use CAs.

## Introduction

### Background

Conversational agents (CAs) are computer programs or artificial intelligence techniques that interact with users through natural language processing [1]. The last decade has witnessed the rapid development of CAs in numerous websites and applications. Such agents are capable of dealing with complex information, delivering more interactive messages, and responding to users in a humanized manner, which benefits a variety of fields such as e-commerce, customer service, web-based education, and health care [2]. In particular, health care CAs can help patients with health care–related decision-making and task management in an efficient and low-cost manner [3]. Therefore, they are frequently applied in the areas of older adults’ care, mental health treatment, behavior change interventions, health care education, and web-based diagnosis and follow-up care [4,5].

Despite their decent performance, there are still great challenges in making health care CAs acceptable to patients [6]. First, there is a debate over whether or to what extent the CAs should be like humans. As CAs have been equipped with certain social characteristics, a number of studies argue that it is necessary to make the interactions between CAs and users as similar as possible to those between human beings [2,7]. In contrast, it has been reported that overly humanized agents may lead to feelings of uncanniness and high expectations from users, which can hinder human-agent interactions to some extent [8,9]. Thus, there is a need to understand how humanness can be attributed to these agents. Second, when managing health care–related tasks using CAs, patients need to disclose personal information and rely heavily on the advice provided by CAs to take further action [10,11]. Thus, patients tend to be more sensitive and vulnerable in such situations, which makes the acceptance and adoption behavior of health care CAs significantly different from those of e-commerce or customer service CAs [2,7]. Thus, it is crucial to understand how humanlike attributes influence patients’ perceptions during interactions with health care CAs and how these perceptions impact patients’ trust building and their acceptance of such agents [5].

A typical example of health care CAs is the intelligent guidance CA used for web-based patient triage in mobile medical consultations [12]. The introduction of such CAs provides a promising way to improve clinic-patient communication in mobile medical consultations [3,13,14]. For example, intelligent guidance CAs can automatically evaluate patients’ conditions and redirect them to suitable physicians based on the symptom information and electronic records provided by patients, which is quite helpful in easing the pressure on mobile health (mHealth) systems [12]. Examples of intelligent guidance CAs used in Chinese mobile medical consultation apps are summarized in Multimedia Appendix 1.

Considering that limited research has explored the effects of humanness design on these intelligent guidance CAs, this study aims to investigate how humanlike cues influence patients’ perceived anthropomorphism and social presence of intelligent guidance CAs and to evaluate how these perceptions facilitate their trust-building process and acceptance behavior. To approach the research objectives, we conducted a within-subject web-based experiment in which participants watched video vignettes that described a hypothetical patient consulting with intelligent guidance CAs designed at various anthropomorphic levels. The following three research questions (RQs) were addressed:

1. RQ 1: How do anthropomorphic design cues, that is, visual cues and language cues, influence participants’ perceived anthropomorphism and social presence attributed to CAs?
2. RQ 2: How do the participants’ perceived anthropomorphism and social presence impact their trust building, that is, trust and privacy concerns, toward CAs?
3. RQ 3: How do the participants’ trust building predict their acceptance of CAs, including their satisfaction, intention to disclose information, and intention to continuously use the CAs?

By explicitly investigating the influences of anthropomorphic cues on intelligent guidance CAs, this study can shed light on the design of health care CAs for supporting trust building and facilitating patients’ acceptance of mobile medical consultation services.

### Theoretical Background and Hypothesis Development

#### How Anthropomorphic Cues Elicit Perceived Anthropomorphism and Social Presence

According to the Computers Are Social Actors paradigm, humans tend to apply social rules and expectations automatically and subconsciously when interacting with computer agents, although they are aware that the agent is not a real person [7,15]. Just as we rely on social cues to understand humans during interpersonal communications, it is also vital to design social cues to facilitate human-agent interactions, such as sex, human voice, facial expressions, gestures, language, and social roles [15-17]. Specifically, social cues are generally defined as “cues that trigger subconscious social reactions” [15]. In previous research, social cues was also referred to as cues, signals, humanlike characteristics, or anthropomorphic features [17]. In particular, our study focuses on the social cues that influence users’ social reactions through various types of anthropomorphic and humanness attributes [16,18], and we termed these cues as “anthropomorphic cues.” We investigated the possible effects of anthropomorphic cues on patients’ perception of anthropomorphism and social presence when attentively watching the process of message interaction between a hypothetical patient and a health care CA.

Anthropomorphic cues can be manipulated in various situations to elicit users’ social reactions to computer agents [2,19]. Prior research demonstrated that the agent’s appearance can stimulate users’ social responses, such as perceiving whether a CA is reliable, committed, and connected. For instance, Beun et al [20] reported that the anthropomorphic level of embodied CAs, that is, realistic, cartoon, or absent visualization, has a positive effect on information retention. Kang and Kim [21] investigated the effects of anthropomorphic cues on the interactions between humans and the Internet of Things (ie, everyday objects embodied by the computing devices that enable internet connectivity and data sending and receiving). The results indicated that the presentation of anthropomorphic cues elicits more positive responses by increasing users’ sense of connectedness. Similarly, anthropomorphic visual cues have been reported to influence customers’ psychological, attitudinal, and behavioral aspects in e-commerce and customer service [2,7]. Nevertheless, the debate remains about whether CAs should look like humans. Posard and Rinderknecht [22] suggested that users behave no differently toward partners with and without anthropomorphic visual cues in a standard trust game. In contrast, it has been suggested that users are more likely to share their interests and report a higher level of commitment with computer partners without anthropomorphic visual cues. Another study reported that participants experienced fewer uncanny effects and intense psychophysiological reactions with a text chatbot than with a humanlike avatar [9].

Besides judging CAs’ similarities to humans based on the interface, another way to enhance human-agent interaction is to use humanlike verbal cues. Verbal cues are emphasized in terms of how social cues are created by words [23]. Greetings and farewells, small talk, thanking, and tips and advice are frequently used as verbal cues for CAs in real estate sales, language learning, Facebook Messenger, voice services, and personal assistants [24,25]. On the basis of the taxonomy of social cues proposed by Feine et al [17], greetings and farewells mean that the CA can express the wordings for welcomes and goodbyes, such as “Nice to meet you” and “goodbye”; small talk means the CA can engage in casual conversations, such as “How are you today?”; thanking means the CA can say thankful words to users, such as “Thanks for your answer”; and tips and advice mean that the CA can provide help or give specific suggestions regarding tasks, such as “I will guide you to the destination.” Such verbal cues would make the CA appear more human because they use the written or spoken words that only appear in interpersonal communications [26]. However, the use of verbal cues is much more complicated and challenging than that of visual cues. As language and conversation are the primary but complex skills that humans develop from very early stages of their lives [2,24], more effort is still needed to investigate how to provide humanlike verbal cues to health care CAs.

Although users treat computers as social actors, debate remains about whether the perceptions of CA anthropomorphism occur as part of a mindful or mindless process [7,18]. To be specific, mindlessness means that human beings rely on a social script—one typically used for interpersonal interactions—to interact with computers, such as perceiving the CAs as “likable,” “sociable,” and “friendly” [7,15,18,22], whereas mindfulness means that people interact with computers by consciously and directly perceiving them as humanlike entities [7,15,22,27], such as perceiving the CAs as “fake or natural,” “machine-like or humanlike,” “unconscious or conscious,” and “artificial or lifelike.” Mindlessness has been evidenced by numerous researchers, who suggested that humans tend to “respond to computer agents in the same way they respond to other human beings” because these social responses can be easily triggered by anthropomorphic and humanlike cues [15,28]. However, Nass and Moon [15] denied the possibility of mindless behaviors being exhibited by participants in their experiment because they asserted that most of the participants were mature adults and experienced computer users. By extending Nass and Moon’s [15] research, Kim and Sundar [18] examined whether social responses to computers are enacted in a mindless or mindful manner. The results suggest that human beings would mindfully refuse to perceive a computerized website as a humanlike entity directly (ie, regarding the website as humanlike or lifelike) and mindlessly identify the website as being human indirectly (ie, perceiving the website as personal or likable). However, Araujo [7] argued that denying the perception of CAs as human entities (mindful anthropomorphism) tends to be less relevant when applied to human-agent interaction because this situation enables more tangible and flexible communication between them. The results of their research proved that participants reported a higher level of mindful anthropomorphism when interacting with humanlike agents.

Overall, it is yet to be answered whether patients tend to regard CAs as human beings consciously and mindfully [15] or treat them unconsciously and automatically in almost the same way as interpersonal interactions [2]. Thus, this study aims to examine the possible influences of anthropomorphic cues on patients’ mindful and mindless anthropomorphism. In line with Araujo’s [7] study, the following hypotheses are proposed:

- H1a: Humanlike agent appearance can increase participants’ perception of mindful anthropomorphism.
- H1b: Humanlike verbal cues can increase participants’ perception of mindful anthropomorphism.
- H2a: Humanlike agent appearance can increase participants’ perception of mindless anthropomorphism.
- H2b: Humanlike verbal cues can increase participants’ perception of mindless anthropomorphism.

The presence of anthropomorphic cues on a computer can also elicit a sense of social presence [19]. Specifically, social presence is defined as “the salience of the other in mediated communication and the consequent salience of their interpersonal interactions” [29]. It describes the ability of a computer to convey social cues, aiming to remind users of humanlike attributes on the interface and then to develop a sense of face-to-face interaction [30]. Considering that the mobile medical consultation environment emphasizes interactions between participants and health care CAs, the sense of social presence becomes crucial to ensuring a close distance in human-agent interaction. In this way, users can perceive attitudes, imagination, warmth, and human touch more easily when interacting with health care CAs [10,31]. On the basis of the Modality, Agency, Interactivity, and Navigability (MAIN) model proposed by Kim and Sundar [18], social presence can be afforded by modality (information presentation with text, audio, or video), agency (computers’ appearance, sex, language, voice, or expertise), interactivity (users’ active engagement with the interface and content), and navigability cues (interior design of the interface). Therefore, humanlike attributes such as agent appearance and verbal cues explicitly convey a heuristic of social presence [32]. Thus, the following hypotheses are proposed:

- H3a: Humanlike agent appearance can increase participants’ perception of social presence.
- H3b: Humanlike verbal cues can increase participants’ perception of social presence.

**Figure 1:**
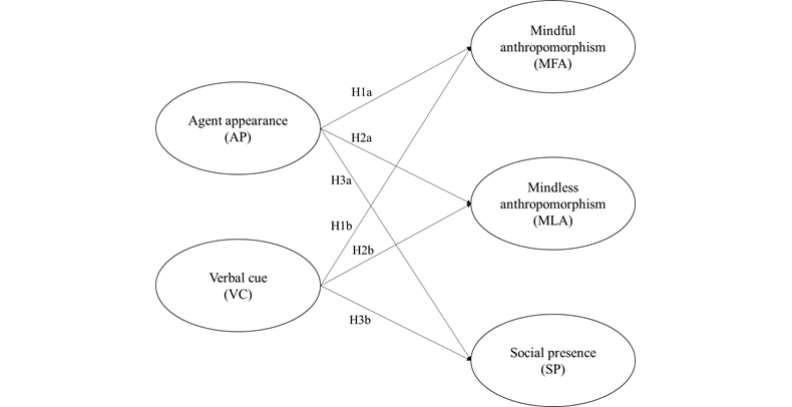
The proposed research model for hypotheses H1a-H3b.

Accordingly, a research model was developed based on H1a-H3b, as shown in Figure 1.

#### How Perceived Anthropomorphism and Social Presence Facilitate Trust Building and Acceptance Behavior

Trust describes a party’s willingness to rely on other parties without considering their monitoring or controlling abilities [33]. There are at least 2 parties involved in relationship development: the trustor, who established the trust, and the trustee, who is to be trusted [5]. In this sense, trust is an important psychological state that develops from interpersonal communication, which is quite helpful in decreasing uncertainty and risk perception during this process [5,34,35]. In medical consultations, patients face high risks to well-being and rely heavily on the advice given by physician teams; therefore, they tend to be more vulnerable and hesitant [36]. Thus, trust building becomes even more important in such situations [10,37]. A trustful relationship is reported to result in an increased willingness to disclose information, better adherence to medical advice, and less fear [10,11,38-40].

In particular, the enhancement of social presence was proven to facilitate trust building by shortening the perceived distance between humans and mHealth apps [5]. As reported by Zhang et al [11], social presence has significant effects on users’ trust in mobile medical consultation apps. Similar evidence can be found in several studies that confirm the influence of social presence on participants’ trust in mHealth apps [32,41-44]. In addition, Kim and Sundar [18] explored the effects of anthropomorphic cues and interactive levels on users’ credibility judgments about health care CAs. They evaluated the direct effect of anthropomorphic cues and interactive levels on participants’ credibility judgments, such as the trustworthiness, reliability, and honesty of the website as well as the mediating effect of social presence on the relationships between these independent variables and information credibility. Although there were some limitations highlighted in this study and they failed to find significant evidence to support all hypotheses, the results emphasized the importance of social presence and anthropomorphism in improving users’ trust in health care CAs. In general, a sense of social presence can shorten the perceived distance between human beings and CAs [5], and perceived anthropomorphism is believed to strengthen their social bonds by increasing CAs’ helpfulness, reliability, commitment, and connectedness [20,21,45], which further encourages the development of trust during the process. Accordingly, we deduce that mindful and mindless anthropomorphism and social presence can positively affect patients’ trust in intelligent guidance CAs, and we hypothesize the following:

- H4: Social presence can positively influence participants’ trust in intelligent guidance CAs.
- H5a: Mindful anthropomorphism can positively influence participants’ trust in intelligent guidance CAs.
- H5b: Mindless anthropomorphism can positively influence participants’ trust in intelligent guidance CAs.

Meanwhile, one of the major hindrances to trust building in mobile medical consultations is privacy risk [46]. With the development of mHealth technologies, a significant amount of personal health data can be automatically obtained and shared with various platforms and systems [47]. In addition, patients also need to disclose sensitive personal information and electronic records from time to time. Generally, privacy concerns refer to a user’s concern about loss of control over the disclosure of personal information to third parties, which may result from unauthorized secondary use of, improper collection of, and improper access to such information as well as errors [48,49]. As CAs are mainly responsible for gathering users’ personal information and points of view, privacy concerns regarding whether a health CA could fulfill its privacy protection responsibilities may become a major factor affecting users’ trust beliefs. Increased privacy concerns may hamper users’ trust in CAs [48,50,51], especially in terms of their cognitive and emotional trust [48]. Thus, we hypothesize the following:

- H6: The participants’ trust in intelligent guidance CAs can be negatively influenced by privacy concerns.

Recent studies have demonstrated that a sense of social presence can reduce users’ privacy concerns [11,52]. The reason may also be that social presence can help shorten the distance between the technology and the user; thus, it can relieve users’ anxiety and uncertainty in web-based environments [53]. For instance, Zhang et al [11] suggested that the social presence of interface and interaction can help reduce patients’ privacy concerns regarding mobile medical consultations. Hong et al [52] reported that social presence is one of the strongest factors that decreases users’ internet privacy concerns. However, Sohn [51] conducted 2 web-based experiments to investigate how the mere presence of messaging services influences users’ privacy concerns. The results revealed that social presence can induce users’ perceptions of being watched, resulting in privacy concerns. To conclude, there is little discussion regarding the effects of social presence on health care CAs. Although the debate over the influence of social presence on privacy concerns is still ongoing, we deduced that a sense of social presence can reduce users’ privacy concerns, based on a majority of established studies [11,52]. The hypothesis is as follows:

- H7: Participants’ privacy concerns about intelligent guidance CAs can be negatively influenced by social presence.

In addition, previous studies demonstrated that the degree of anthropomorphism has a significant influence on users’ perceived risks [54]. Although people tend to show a generally positive attitude toward humanized agents, there are some concerns about the extent to which these agents should be anthropomorphized. As also implied by the aforementioned hypotheses, CAs are usually asked to collect personal and sensitive information, which increases the privacy concerns of users. Therefore, on the one hand, humanized CAs can easily elicit individuals’ feelings of familiarity, interests, and comfort through their presented human characteristics. For example, Ischen et al [50] reported that a humanlike chatbot could lead to fewer privacy concerns in a commercial context such as product recommendations. On the other hand, a humanized CA may make the user feel more vulnerable to improper information exchange or unauthorized secondary use of personal information because CAs have many humanlike features. In other words, users may easily feel that humanized CAs have become co-owners of their personal information; therefore, they tend to decrease the time of information disclosure to reduce any uncertainty brought about by humanized CAs [45,55]. Ha et al [45] found that intelligent web-based assistants with anthropomorphic cues lead to greater privacy concerns because users’ fear about the improper disclosure of information outweighs the possible benefits of familiarity. Xie et al [56] indicated that anthropomorphic websites induce a higher level of privacy concerns for consumers who have a low need for interaction and who experience social exclusion, which suggests that the incorporation of anthropomorphic cues needs to consider users’ individual characteristics. Therefore, we propose that patients’ mindful and mindless anthropomorphism can significantly increase their privacy concerns, as follows:

- H8a: Participants will have greater privacy concerns when they perceive a higher level of mindful anthropomorphism.
- H8b: Participants will have greater privacy concerns when they perceive a higher level of mindless anthropomorphism.

In conclusion, the aforementioned perceptual attributes combine to influence an individual’s acceptance of health care CAs. For example, previous studies have suggested that privacy concerns are an important predictor of users’ intention to disclose information [57,58]. Therefore, it is assumed that privacy concerns negatively affect participants’ intention to disclose information to health care CAs. Additionally, trust between users and CAs plays a crucial role in predicting users’ behavioral intentions regarding web-based medical consultations. First, similar to privacy concerns, a certain level of trustworthiness can help decrease the feelings of uncertainty or complications when individuals interact with agents, which can further improve their willingness to disclose personal information [37-39,59]. Second, if individuals believe that the CA is capable of protecting their personal information and providing reliable services, they are more likely to have a higher level of satisfaction, which encourages further intention to continually use health care CAs [11,37,39,46,60]. Furthermore, following the relevant research regarding the Technology Acceptance Model [61], users’ attitudes, which are defined as their positive or negative feelings when interacting with a health care CA, are also believed to influence their intention to continuously use the health care CA. Therefore, we propose the following hypotheses based on the aforementioned assumptions:

- H9: Privacy concerns can negatively influence participants’ intention to disclose information.
- H10: Trust can positively influence participants’ intention to disclose information.
- H11: Trust can positively influence participants’ satisfaction with intelligent guidance CAs.
- H12: Trust can positively influence the participants’ intention to continuously use intelligent guidance CAs.
- H13: Participants’ satisfaction can positively influence their intention to continuously use intelligent guidance CAs.

**Figure 2:**
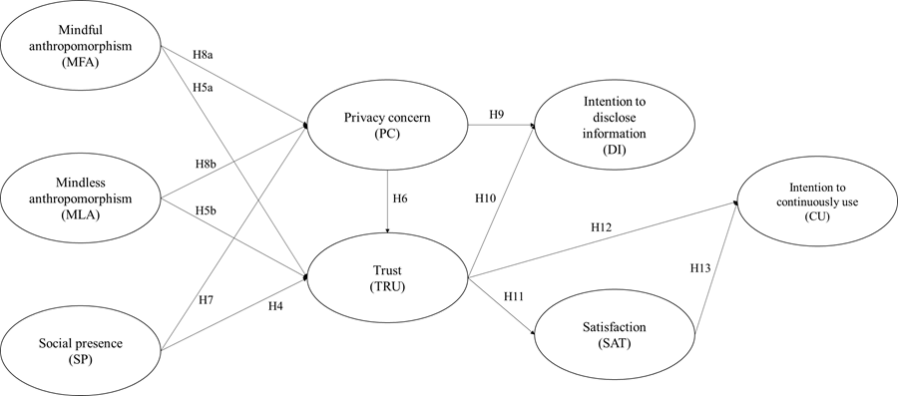
The proposed research model for hypotheses H4-H13.

Thus, a research model was developed based on hypotheses H4-H13, as shown in Figure 2.

## Methods

### Overview of the Web-Based Experiment

A 3×2 within-subject web-based experiment was conducted using one of the largest web-based survey systems in China, Wen Juan Xing, in May 2022. The video vignette methodology was used to evaluate patients’ perceptions and acceptance of intelligent guidance CAs, in which 6 hypothetical scenarios were developed to describe the web-based diagnosis processes guided by various intelligent guidance CAs. This method has been successfully applied to examine users’ perceptual and attitudinal attributes in human-robot interaction studies [62-65]. Considering that this study is an initial step in exploring possible design directions for intelligent guidance CAs, the video vignette seems to be an effective method.

### Participants’ Recruitment

Participants were required to be older than 18 years. They were potential users of intelligent guidance CAs with previous use experience of mobile medical consultation services, that is, they had previously sent text-, picture-, or voice-based messages or used voice and video chats to consult with web-based doctors. The sampling restriction is enforced because we mainly explored the anthropomorphism perceptions, trust, and acceptance of health care CAs among mobile medical consultation users, which was similar to previous research [66-70]. After sending out 496 questionnaires, we obtained 170 questionnaires by setting a screening question to ask whether the potential participants were current users of mobile medical consultation services (response rate: 34.3%). Subsequently, by checking the attention filter questions, 103 valid samples were collected.

### Informed Consent

Before the study, each participant was informed about the need to consent to participate in this research. In the consent form, participants were required to indicate whether the experimental procedure was fully explained and whether the benefits and risks involved in this research were well understood. Participants were notified that they have the right to question any part of the procedure and can withdraw at any time without penalty. They were also instructed that the information obtained from this research would be used in future research and published, but their rights to privacy would be retained, that is, their personal details would not be shared (Informed Consent). After completing the web-based experiment, each participant received a reward of ¥10 (US $1.50) for their participation.

### Ethics Approval

This study was approved by the School of Design, The Hong Kong Polytechnic University (HSEARS20210316004).

### Experiment Design, Material, and Stimulus

#### Overview of Experiment Design

To test the research hypotheses, the anthropomorphic cues were manipulated in a 3×2 within-subject factorial experiment, with the factors of agent appearance (high vs medium vs low anthropomorphic level) and verbal cues (humanlike verbal cues vs machine-like verbal cues) as the within-subject variables.

Instead of inviting participants to interact with the intelligent guidance CAs directly, this study chose the video recording of dynamic message interaction as the experimental stimulus owing to 2 reasons. On the one hand, the video recording can elicit the participants’ interaction experiences in a vivid way by describing a dynamic message interaction process. On the other hand, it can assure higher reliability and fluency by eliminating the possible confounding effects of usability problems or technical issues that may be induced by a free interactive experience.

**Figure 3:**
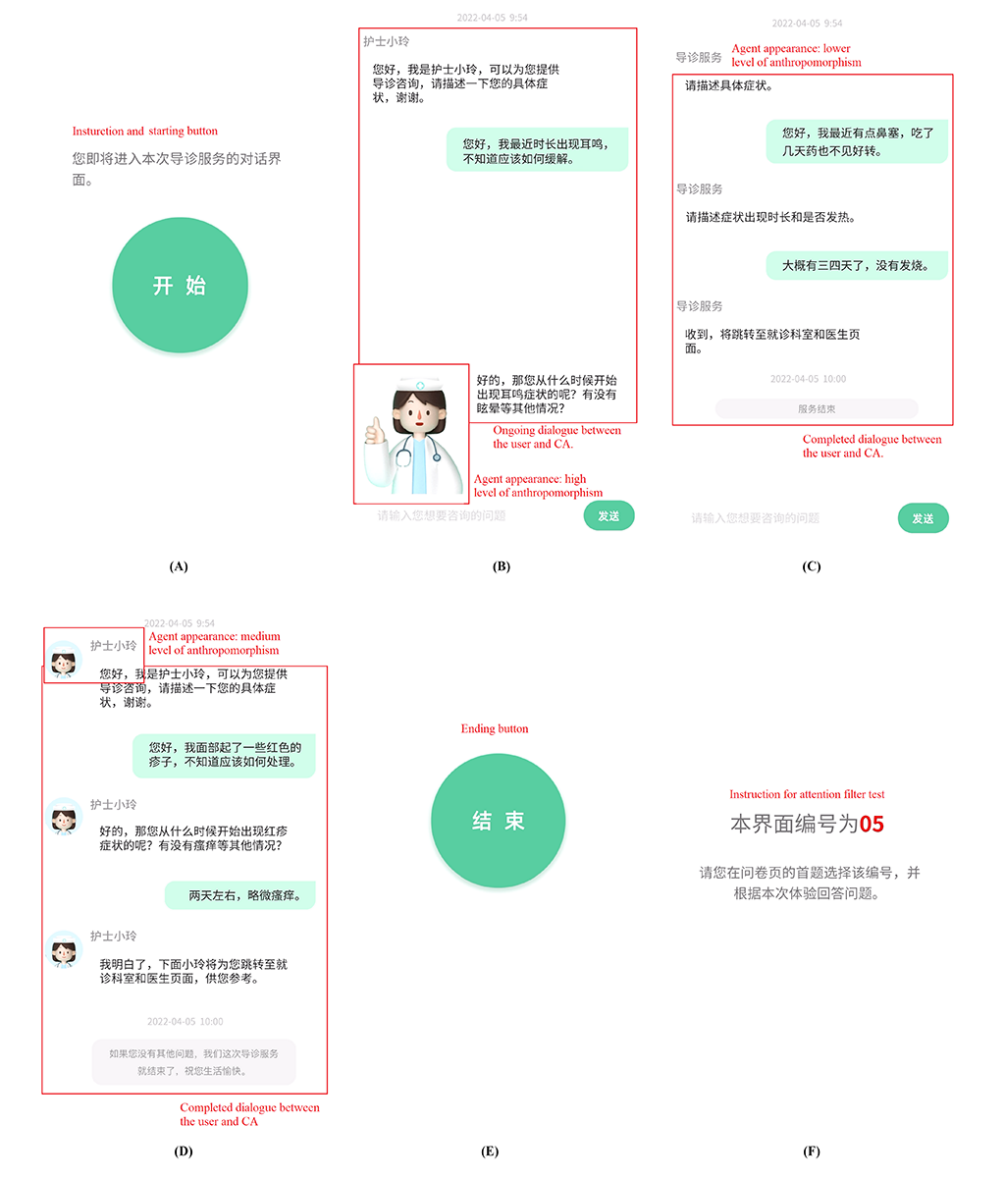
Screenshots of 1 video example used as the experimental stimulus: (A) staring, (B) ongoing dialogue, (C and D) completed dialogue, (E) ending, and (F) attention filter question.

For each experimental condition, a high-fidelity prototype of the intelligent guidance page was constructed using Figma software (Adobe). The dynamic process of message interaction between the intelligent guidance CA and a hypothetical patient was recorded as the video vignette. Some screenshots of the videos are shown in Figure 3. The format of the intelligent guidance page was modified from the styles of several of the highest-ranked mHealth apps in the Chinese app market, including Ping An Health (Ping An Healthcare And Technology Company Limited), Ali Health (Alibaba Health Information Technology Limited), Weixin Smart Hospital(Tencent Holdings Limited), and Chunyu Doctor (Spring Rain Software). In particular, they showed a dynamic message interaction process, from a hypothetical patient starting to disclose information about his or her symptoms to an intelligent guidance CA claiming that it would provide a proper physician for him or her. Five sentences were involved in the message interaction process. Three of them were initiated by the CA, including 1 sentence asking about the symptoms, 1 sentence pressing for more details, and 1 sentence providing suggestions. The other two were initiated by the hypothetical patient, including 1 sentence describing the symptoms and 1 sentence explaining more details. During the message interaction process, the dialogues between the hypothetical patient and intelligent guidance CA appeared one by one. To simulate a vivid medical consultation experience and eliminate possible emotional responses for the participants, we selected 6 mild symptoms to describe in the video vignettes: stuffy nose, cough, rash, tinnitus, and abdominal discomfort. On the basis of the content length, the interval between the appearance of each sentence was set to 4 to 7 seconds. In total, 6 video vignettes were developed; an example is presented in Multimedia Appendix 2. Each video vignette lasted for approximately 50 to 60 seconds.

To overcome the problem of order effects, the method of counterbalancing was used, and participants were randomly assigned to the experimental conditions. Video vignettes were inserted into the experimental questionnaires. At the end of each video, the number of experimental conditions was briefly shown for 3 seconds. The participants were asked to watch the videos and were encouraged to attentively experience the interaction scenarios. After that, an attention filter question was asked about the number of experimental conditions to ensure the quality of the experimental data. The participants who answered the wrong number for any of the experimental conditions were believed to be inattentive, and their questionnaires were deleted accordingly.

#### Agent Appearance

**Figure 4:**
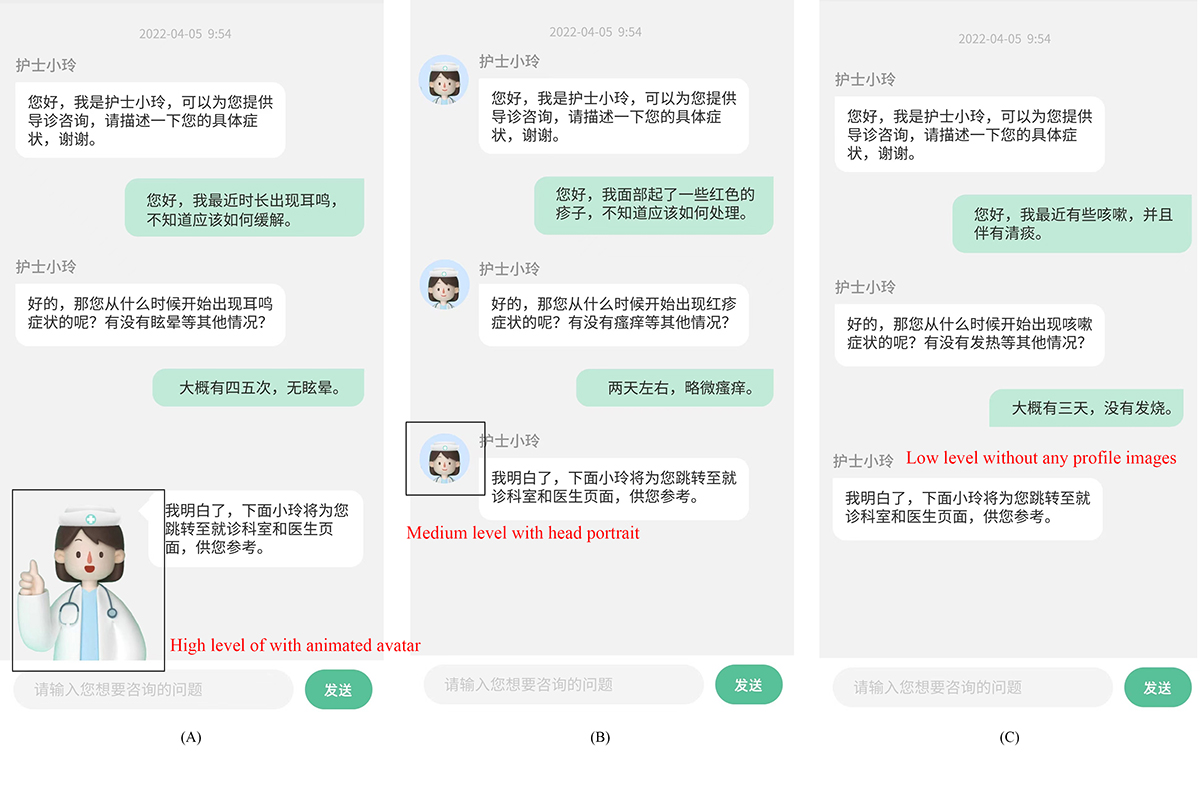
Examples of experimental manipulation of 3 anthropomorphic levels of agent appearance: the (A) high level with animated avatar, (B) medium level with head portrait, and (C) low level without any profile images.

This study manipulated the anthropomorphic level of agent appearance based on previous studies that emphasized the importance of the degree of human likeness and facial expressions [17]. Therefore, 3 anthropomorphic levels of agent appearance were designed for this experiment (Figure 4). At the high anthropomorphic level, the intelligent guidance CA appeared as an animated, humanlike, cartoon avatar. The avatar displayed some simple facial expressions, such as smiles, winks, and mouth movements, while chatting. At the medium anthropomorphic level, the participants were exposed to an intelligent guidance CA with a head portrait, which was a static image of a humanlike avatar. At the low anthropomorphic level, the intelligent guidance CA did not have any profile pictures or images.

#### Verbal Cues

**Figure 5:**
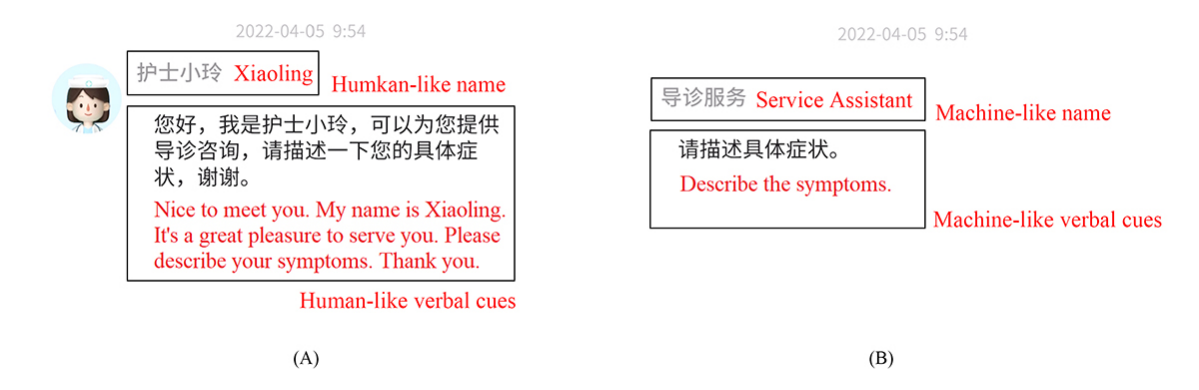
Example of experimental manipulation of 2 anthropomorphic levels of verbal cues: the level of (A) humanlike verbal cues and (B) machine-like verbal cues.

The experimental manipulation of verbal cues was also based on the methods used in previous studies [2], as shown in Figure 5. In the humanlike condition, the intelligent CA had a human name (Xiaoling), and it could express the wordings of self-introduction, greetings, farewells, thanking, and tips and advice, such as “Nice to meet you,” “I’m Xiaoling,” “May I help you?,” “Goodbye,” “Have a nice day,” and “I will guide you to a recommended physician.” In contrast, at the level with machine-like verbal cues, it had a nonhuman name (service assistant), and it did not include any humanlike language or conversation. Instead, it used machine-like dialogue cues, such as “start” and “quit.” In addition, to avoid possible confusion regarding the identity of the intelligent CA, the participants were informed before the experiment that the intelligent guidance CA was operated by a chatbot rather than a human.

### Measurement

To test the proposed hypotheses and conceptual model, an experimental questionnaire was designed comprising 2 sections (Multimedia Appendix 3). The first section was used to investigate the participants’ demographic information and mobile medical consultation service experience. Specifically, each participant’s age, sex, education level, city of residence, and profession were collected. Their previous use experience with mobile medical consultation services and intelligent guidance was noted, such as the mHealth app where they received the mobile medical consultation services, whether they had ever used intelligent guidance services, the duration of time since they first used mobile medical consultation services, the number of times they had used mobile medical consultation services, the registered departments, and the communication methods used when using mobile medical consultation services.

The second section assessed the participants’ perceived anthropomorphism, social presence, trust building and acceptance behavior. Specifically, the participants’ mindful anthropomorphism was evaluated, using 4 items assessing whether they felt like the intelligent guidance CA was “fake or natural,” “machine-like or humanlike,” “unconscious or conscious,” and “artificial or lifelike” [7,27]. Three items were used to assess the participants’ mindless anthropomorphism by the extent to which the participants agreed that the intelligent guidance CA was “likable,” “sociable,” and “friendly” [7,18]. The perception of social presence was measured by using 4 items adapted from the studies by Zhang et al [11] and Lu et al [71]: “Can you make sense of the attitude of the intelligent guidance CA?,” “Can you imagine how the intelligent guidance CA may look?,” “Is there a sense of human touch to communicate with the intelligent guidance CA?,” and “Is it warm when communicating with the intelligent guidance CA?”

In addition, trust was measured, using 4 items adapted from the studies by Li [37], Guo et al [70], Akter et al [72], McKnight et al [73], and Teo and Liu [74], by asking the participants whether the intelligent guidance CA was “trustworthy,” could “provide reliable information,” could “keep promises and commitments,” and whether it had “met their expectations.” Privacy concerns were assessed, using 4 items adapted from the studies by Guo et al [70] and Featherman and Pavlou [75], by asking the participants the extent to which the use of intelligent guidance CA could “make them lose control over their privacy of information,” “cause any privacy problems,” “lead to a loss of privacy because their personal information could be used without their knowledge,” or “make others take control of their information.” Finally, participants’ acceptance of intelligent guidance CAs was investigated through their satisfaction, intention to disclose information, and intention to continuously use intelligent guidance CAs, which were derived from the studies by Li [37], Chen et al [76], Sharma and Crossler [77], and Zhang et al [58]. Specifically, the participants’ satisfaction with the intelligent guidance CA was measured by whether they thought it was a good idea to use it and whether they thought it was wise to use it, as well as whether they liked the idea of making use of it. Their intention to disclose information was evaluated by asking whether they are likely, plan to, or intend to provide their personal information and health information when using the intelligent guidance CA. The intention to continue use was finally reflected by whether the participants intend to, believe, and plan to use the intelligent guidance CA in the future.

Each item of the experimental questionnaire was measured using a 7-point Likert scale, ranging from 1 (strongly disagree) to 7 (strongly agree). Two local experts with extensive user research experience helped validate the instrument items and modified some wording in the Chinese version of the questionnaire. After that, the experimental questionnaire was pilot-tested with 5 potential patients from a variety of age groups and educational backgrounds and then further revised in terms of the written language and question sequence based on their feedback.

### Procedure

At the beginning of the experiment, the participants were asked about their demographic information (ie, age, sex, education level, city of residence, and profession) and mobile medical consultation service experience (ie, the mHealth app used, experience of using intelligent guidance services, the duration of use, the number of times the service was used, the registered departments, and the communication methods). The participants were then asked to watch 6 videos of stimuli materials one by one. The order of the stimuli materials for each participant was randomly assigned. After watching each video, they were required to report their perceived anthropomorphism, social presence, trust, privacy concerns, and acceptance behavior.

### Data Analysis

A descriptive analysis was first conducted on the participants’ demographic data (ie, age, sex, education level, city of residence, and profession) and previous use behavior with mobile medical consultation services (ie, the mHealth app where they received the mobile medical consultation services, whether they had ever used intelligent guidance services, the duration of time since they first used mobile medical consultation services, the frequency of using mobile medical consultation services, the registered departments, and the communication methods when using mobile medical consultation services).

A *Z* test was then used to test the data normality of the mean scores in terms of the participants’ mindful anthropomorphism, mindless anthropomorphism, and social presence, as this can overcome the limitations of using the skewness or kurtosis (excess) of data to indicate normality for a small-to-moderate sample size (ie, n<300). Specifically, *Z* scores were calculated by dividing the skewness values or excess kurtosis values by their SEs. The results indicated that the abovementioned data were normally distributed, with an absolute *Z* value of +3.29 and −3.29 [78]. Therefore, a 2-way repeated measures ANOVA was used to compare the effects of agent appearance (high, medium, and low anthropomorphic levels) and verbal cues (humanlike and machine-like) on the participants’ perceptions of mindful and mindless anthropomorphism as well as social presence to test the first 3 hypotheses (H1-H3). In each case, the assumption of sphericity was tested, and all effect sizes were calculated. An α level of .05 was used for statistical analysis. In addition, G*Power 3.1.9.4 software (Universität Düsseldorf) was used to calculate the sample size for ANOVA analysis. The results suggested that 20 observations are required to achieve a statistical power of 80% for a medium effect size of 0.25 (with a 5% probability of error). Thus, the valid samples recruited in this study fulfilled the sample size requirement for the ANOVA analysis.

Structural equation modeling was used to test the subsequent hypotheses (H4-H13) proposed in this study. The partial least squares (PLS) method was used to test the research model because it is more suitable for testing small samples [79] and is more appreciated for evaluating theoretical models developed at an early stage [80]. In addition, by calculating the loadings and weights of construct indicators, the PLS method is suitable for assessing the causal relationships among the different stages and layers of model constructs [80]. Therefore, it was deemed ideal for this study, and the scores for all the items regarding mindful anthropomorphism, mindless anthropomorphism, social presence, trust, privacy concerns, satisfaction, intention to disclose information, and intention to continuously use intelligent guidance CAs were calculated using the PLS method. The sample size for PLS analysis was also calculated using G*power 3.1.9.4 software. The results suggested that 48 observations are required to achieve a statistical power of 80% for detecting *R*^2^ values of at least 0.25 (with a 5% probability of error), which is also lower than the valid sample that we actually recruited. The item-item correlation matrix of acceptance predictors was shown in Multimedia Appendix 4.

Composite reliability was used to test the reliability of the constructs formulated in the experimental questionnaires. The composite reliability values in this study ranged from 0.763 to 0.929, which were higher than the suggested cutoff value of 0.700 [37,80]. The average variance extracted (AVE) was used to compare the consistency between the instrument measurements used in this study with those used in previous studies. The AVE values ranged from 0.704 to 0.860, which also exceeded the suggested accepted value of 0.500 [80]. Therefore, the results indicated that the constructs and measurements used in this study were well constructed and reliable. In addition, convergent validity was measured based on the item loading, and any loading <0.700 was considered insufficient to measure the instrument construct [73,80]. As indicated by the results, all the construct items had a loading value of >0.700, ranging from 0.763 to 0.943. In other words, the instrument constructs formulated in the experimental questionnaire had good convergent validity. Discriminant validity was further examined to check the similarity of measurements between various construct pairs. Discriminant validity is ensured when the square root of the AVE for a construct is greater than the correlations between the construct and other constructs in the research model [80]. In this study, the square roots of all AVEs were larger than the correlations between the construct and other constructs, suggesting favorable discriminant validity. The means, SDs, reliability, convergent validity, and discriminant validity of all constructs in the measurement model are presented in Multimedia Appendix 5.

## Results

### Participants and Use Experience

A total of 103 participants (37 male participants and 66 female participants) participated in the web-based experiment. Their average age was 30.5 (SD 5.49; range 19-47) years. Participants reported a range of education levels, ranging from high school to postgraduate and above.

Regarding the participants’ previous use behavior with mobile medical consultation services, 34.95% (36/103) had adopted mobile medical consultations for 2 to 3 years, followed by those who had adopted mobile medical consultations for 1 to 2 years, >3 years, and <1 year. Participants also reported the number of times they used mobile medical consultation services. Except for 2 participants who were unsure about the number of times they had used such services, more than half of the participants had used mobile medical consultations for ≥5 times. In particular, 96.1% (99/103) of the participants had used web-based patient triage in mobile medical consultation services, which indicated that web-based patient triage in mobile medical consultations is a crucial and necessary step in mobile medical consultations. Furthermore, participants indicated the use of the following web-based hospital departments, with frequencies ranging from high to low: dermatology, internal medicine, surgery, stomatology, ophthalmology, obstetrics and gynecology, pediatrics, orthopedics, and others (ie, traditional Chinese medicine). Regarding communication methods with physicians through mobile medical consultations, 98.1% (101/103) of the participants used text messaging, followed by those who used image messages, voice messages, audio chat, video messages, telephone calls, chat groups in QQ (Tencent Holdings Ltd) or WeChat (Tencent Holdings Ltd), and video chat. Participants’ demographic information and previous use behavior with mobile medical consultation are shown in Table 1.

**Table 1:** Participants’ demographic information and previous use behavior with mobile medical consultation (MMC; N=103).

		Values, n (%)
**Sex**		
	Male	37 (35.9)
	Female	66 (64.1)
**Age (years)**		
	18-25	16 (15.5)
	26-35	72 (69.9)
	36-45	14 (13.6)
	46-55	1 (1.0)
**Education level**		
	High school	1 (1.0)
	Undergraduate	93 (90.3)
	Postgraduate and above	9 (8.7)
**Duration (years) of using MMC services**		
	<1	20 (19.4)
	1-2	26 (25.2)
	2-3	36 (34.9)
	>3	21 (20.4)
**Number of times MMC services were used**		
	1-2	2 (1.9)
	2-5	38 (36.9)
	>5	61 (59.2)
	Not sure	2 (1.9)
**Registered web-based hospital departments**		
	Dermatology	64 (62.1)
	Internal medicine	58 (56.3)
	Surgery	43 (41.8)
	Stomatology	38 (36.9)
	Ophthalmology	30 (29.1)
	Obstetrics and gynecology	20 (19.4)
	Pediatrics	14 (13.6)
	Orthopedics and others	12 (11.7)
**Communication methods through MMCs**		
	Text messaging	101 (98.1)
	Image messages	86 (83.5)
	Voice messages	55 (53.4)
	Audio chat	35 (34)
	Video messages	27 (26.2)
	Telephone calls	26 (25.2)
	Chat groups in QQ or WeChat	25 (24.3)
	Video chat	13 (12.6)

### ANOVA Results for Manipulation Checking

#### Overview

The results of the means and SDs of the participants’ evaluations of mindful anthropomorphism, mindless anthropomorphism, and social presence are shown in Table 2.

**Table 2:** Means and SDs of perception evaluations of mindful anthropomorphism, mindless anthropomorphism, and social presence (N=103).

Within-subject variables (agent appearance and verbal cues)		Perceived anthropomorphism and social presence, mean (SD)		
		Mindful anthropomorphism	Mindless anthropomorphism	Social presence
**High anthropomorphic level**				
	Humanlike	5.01 (1.53)	5.81 (0.76)	4.20 (0.72)
	Machine-like	4.12 (1.78)	4.98 (1.36)	3.55 (1.16)
**Medium anthropomorphic level**				
	Humanlike	4.82 (1.58)	5.49 (0.92)	3.95 (0.90)
	Machine-like	3.73 (1.78)	4.52 (1.45)	3.20 (1.21)
**Low anthropomorphic level**				
	Machine-like	4.65 (1.62)	5.50 (0.90)	3.96 (0.90)
	Humanlike	3.43 (1.79)	4.20 (1.52)	2.98 (1.22)

#### Agent Appearance

The Mauchly test indicated that the data on perceived mindful anthropomorphism for different levels of agent appearances failed to meet the assumption of sphericity (*χ*^2^_2_=16.1; *P*<.001). Thus, the df values were corrected using Huynh and Feldt estimates of sphericity (Ɛ=0.89). The results showed a significant main effect of agent appearance on participants’ perceived mindful anthropomorphism (*F*_1.77,180.60_=12.241; *P*<.001). The data on perceived mindless anthropomorphism and social presence for different levels of agent appearances met the assumption of sphericity (*χ*^2^_2_=4.3; *P*=.12 and *χ*^2^_2_=4.6; *P*=.10). The results confirmed the significant and positive main effects of agent appearance on participants’ mindless anthropomorphism (*F*_2,204_=23.450; *P*<.001) and social presence (*F*_2,204_=17.105; *P*<.001). H1a, H2a, and H3a were supported.

Specifically, Bonferroni post hoc test results indicated that participants perceived a statistically significant higher level of mindful anthropomorphism when facing an agent with a high anthropomorphic level than when facing an agent with a medium (4.57 vs 4.27; *P*=.01) or low (4.57 vs 4.04; *P*<.001) anthropomorphic level. In addition, participants perceived a significantly higher level of mindful anthropomorphism when facing an agent with a medium anthropomorphic level than when facing an agent with a low anthropomorphic level (4.27 vs 4.04; *P*=.04). In other words, participants generally believed that agents with a high level of anthropomorphism tended to be more natural, human like, conscious, and lifelike.

In addition, based on the results of the Bonferroni post hoc test, a significantly higher level of mindless anthropomorphism was reported in the high anthropomorphic level of agent appearance than in the medium (5.39 vs 5.01; *P*<.001) or low (5.39 vs 4.85; *P*=.003) anthropomorphic level, which indicated that participants perceived agents with high levels of anthropomorphism as more likable, sociable, and friendly. Nevertheless, there was no significant difference in mindless anthropomorphism between the medium and low anthropomorphic levels of agent appearance.

Similarly, the Bonferroni post hoc test indicated that participants perceived a higher level of social presence with an agent with a high anthropomorphic level of agent appearance than with a medium (5.19 vs 4.83; *P*=.01) or low (5.19 vs 4.72; *P*<.001) anthropomorphic level. This implied that participants could better make sense of the attitudes of the CAs and imagine how they may look as well as perceive a sense of human touch and warmth when communicating with the CAs with a high anthropomorphic level of agent appearance. However, no significant difference in social presence was found between the medium and low anthropomorphic levels of agent appearance.

#### Verbal Cues

The results also reported significant and positive main effects of verbal cues on participants’ mindful anthropomorphism (*F*_1,102_=55.896; *P*<.001), mindless anthropomorphism (*F*_1,102_=95.686; *P*<.001), and social presence (*F*_1,102_=81.187; *P*<.001), which supported H1b, H2b, and H3b. Overall, the participants perceived a higher level of mindful anthropomorphism facing CAs with humanlike verbal cues (4.83 vs 3.76) and felt that they were more natural, humanlike, conscious, and lifelike than those with machine-like verbal cues. Participants also reported a higher level of mindless anthropomorphism (5.60 vs 4.57) facing CAs with humanlike verbal cues and felt they were more likable, sociable, and friendly than those with machine-like verbal cues.

#### The Interaction Between the Agent’s Appearance and Verbal Cues

**Figure 6:**
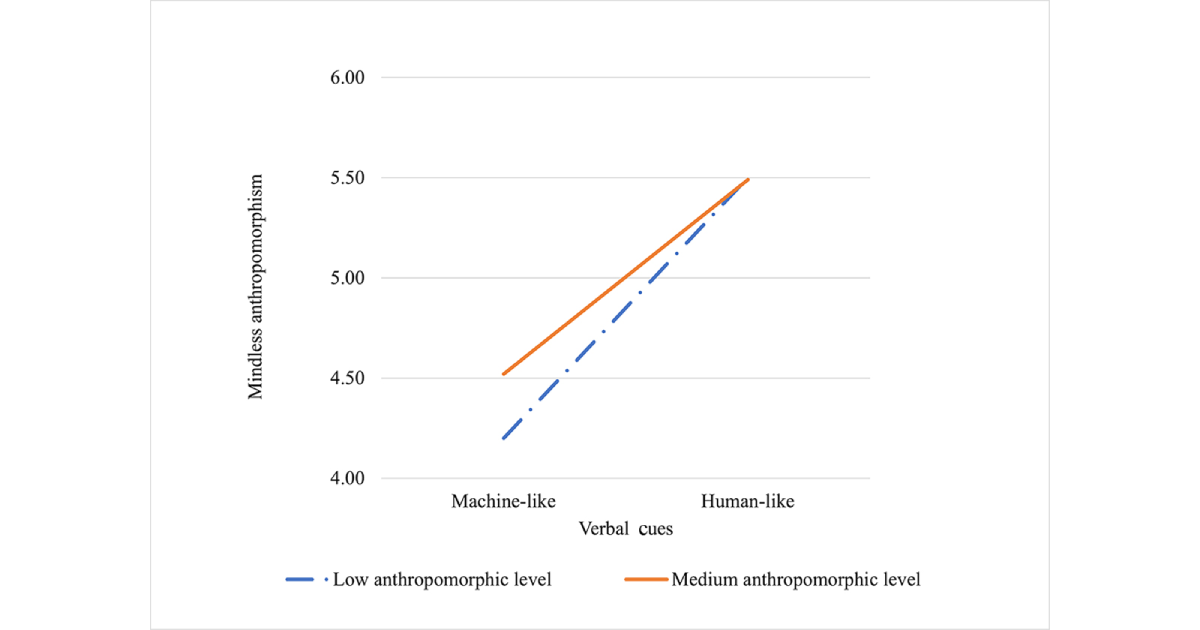
Mean mindless anthropomorphism for machine-like and humanlike verbal cues at low and medium anthropomorphic levels.

There was a significant interaction between the agent’s appearance and verbal cues (*F*_2,204_=5.670; *P*=.004) when the agent’s appearance was at low and medium levels of anthropomorphism, as shown in Figure 6. It showed that when facing CAs with machine-like verbal cues, the participants perceived a higher level of mindless anthropomorphism for the agent that appeared with a medium anthropomorphic level than that with a low anthropomorphic level (4.52 vs 4.20). However, when facing CAs with humanlike verbal cues, they perceived a nearly equal level of mindless anthropomorphism, regardless of whether the agent had a medium or low anthropomorphic appearance (5.49 vs 5.50). In other words, when the agent’s appearance was at a low or medium anthropomorphic level, its effects may become less significant if the health care CAs were designed with humanlike verbal cues. Nonetheless, no other significant interaction effect was revealed from the results.

Table 3 summarizes the effects of agent appearance and verbal cues on the participants’ mindful anthropomorphism, mindless anthropomorphism, and social presence.

**Table 3:** Results of hypothesis testing based on ANOVA tests.

Hypotheses and supported (yes or no)		Findings	Pairwise comparisons between anthropomorphism levels	
			Pairs	Significant
**H1a (AP^a^→MFA^b^)**				
	Yes	Humanlike AP can increase participants’ perception of MFA.	High level versus medium level	Yes (*P*=.01)
	Yes	Humanlike AP can increase participants’ perception of MFA.	High level versus low level	Yes (*P*<.001)
	Yes	Humanlike AP can increase participants’ perception of MFA.	Medium level versus low level	Yes (*P*=.04)
**H1b (VC^c^→MFA)**				
	Yes	Humanlike VC can increase participants’ perception of MFA.	Humanlike versus machine-like	Yes (*P*<.001)
**H2sa (AP→MLA^d^)**				
	Yes	Humanlike AP can increase participants’ perception of MLA.	High level versus medium level	Yes (*P*=.003)
	Yes	Humanlike AP can increase participants’ perception of MLA.	High level versus low level	Yes (*P*<.001)
	Yes	Humanlike AP can increase participants’ perception of MLA.	Medium level versus low level	No (*P*=.38)
**H2b (VC→MLA)**				
	Yes	Humanlike VC can increase participants’ perception of MLA.	Humanlike versus machine-like	Yes (*P*<.001)
**H3a (AP→SP^e^)**				
	Yes	Humanlike AP can increase participants’ perception of SP.	High level versus medium level	Yes (*P*=.01)
	Yes	Humanlike AP can increase participants’ perception of SP.	High level versus low level	Yes (*P*<.001)
	Yes	Humanlike AP can increase participants’ perception of SP.	Medium level versus low level	No (*P*=.54)
**H3b (VC→SP)**				
	Yes	Humanlike VC can increase participants’ perception of SP.	Humanlike versus machine-like	Yes (*P*<.001)

^a^AP: agent appearance.

^b^MFA: mindful anthropomorphism.

^c^VC: verbal cue.

^d^MLA: mindless anthropomorphism.

^e^SP: social presence.

### PLS Results for Hypotheses Testing

**Figure 7:**
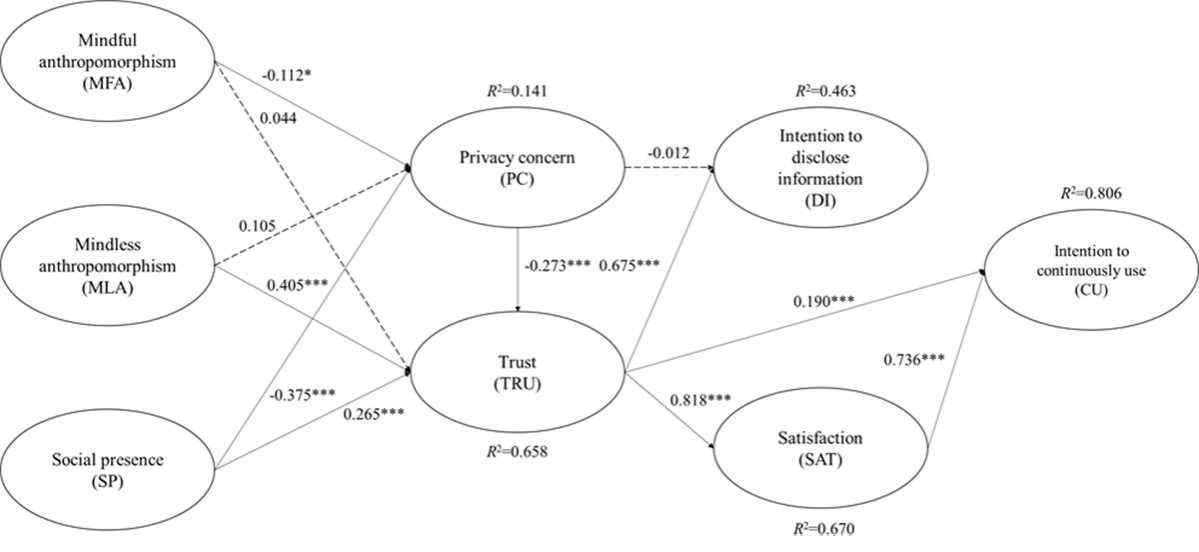
Partial least squares results for the research model. *P<.05; ***P<.001.

A structural model was developed to ascertain any existing causal relationships by measuring their significance levels and path coefficients, using the bootstrap method (with 5000 subsamples). The PLS results are shown Figure 7 and Table 4. The coefficients of determination (*R^2^*) were used to explain the percentage of variance contributed by the independent variable proposed in the research model. The variance accounted for (VAF) value was calculated to evaluate the mediation effects in the structural model [79]. In particular, the *R^2^* values of participants’ intention to continuously use CAs, satisfaction, and trust were comparably high. The amount of explained variance in the intention to disclose information can be considered moderate. Although the *R^2^* value of privacy concerns was slightly lower than the suggested coefficient of determination of 0.190 [81], it was still regarded as acceptable owing to the exploratory nature of our research.

In total, the participants’ perceptions of mindless anthropomorphism and social presence accounted for 65.8% of the variance in their trust in intelligent guidance CAs (*R^2^*=0.658); 14.1% of the variance in privacy concerns was explained by the participants’ perceived social presence and mindful anthropomorphism (*R^2^*=0.141); participants’ trust in CAs could explain 46.3% of the variance in their intention to disclose information (*R^2^*=0.463) and 67% of the variance in their level of satisfaction (*R^2^*=0.670); and 80.6% of the variance in participants’ intention to continuously use CAs could be explained by their trust and satisfaction with CAs (*R^2^*=0.806).

For hypothesis testing, the participants’ perceived social presence (β=.265; *t*_312_=4.314) and mindless anthropomorphism (β=.405; *t*_312_=7.145) had significant positive influences on their trust in CAs. Simultaneously, the effect of perceived social presence on trust was partially mediated by the participants’ privacy concerns (VAF=27.88%). The effect of perceived mindful anthropomorphism on trust was fully mediated by the participants’ privacy concerns, and no direct effect was found between mindful anthropomorphism and trust. At the same time, privacy concerns were found to be negatively influenced by the participants’ perceptions of social presence (β=−.375; *t*_312_=4.494) and mindful anthropomorphism (β=−.112; *t*_312_=1.970), whereas no effects were found in their perceptions of mindless anthropomorphism. Therefore, H4, H5b, and H7 were supported, and H5a and H8b were rejected. Although a marginally significant effect was found between mindful anthropomorphism and privacy concerns, it was negative. Thus, H8a was rejected. Furthermore, we found that participants’ privacy concerns significantly affected their trust in CAs (β=−.273; *t*_312_=9.558), and H6 was thus supported.

As for the participants’ acceptance of CAs, the results indicated that trust in CAs could positively and significantly influence their intention to disclose information (β=.675; *t*_312_=21.163), intention to continuously use CAs (β=.190; *t*_312_=4.874) and satisfaction (β=.818; *t*_312_=46.783). The effect of privacy concerns on participants’ intention to disclose information was found to be fully mediated by trust, and no direct explanatory relationship was reported between them. Thus, H10, H11, and H12 were supported, whereas H9 was rejected. In addition, it was found that the participants’ level of satisfaction could also positively influence their intention to continuously use CAs (β=.736; *t*_312_=19.964) and partially mediate the effect of trust on the participants’ intention to continuously use CAs (VAF=76.01%). Thus, H13 was supported.

**Table 4:** Results of hypothesis testing based on partial least squares tests.

Hypotheses	Path	Path coefficient	*t* test (*df*)	*P* value	Supported
H4	SP^a^→TRU^b^	0.265	4.314 (312)	<.001	Yes
H5a	MFA^c^→TRU	0.044	1.180 (312)	.24	No
H5b	MLA^d^→TRU	0.405	7.145 (312)	<.001	Yes
H6	PC^e^→TRU	−0.273	9.558 (312)	<.001	Yes
H7	SP→PC	−0.375	4.494 (312)	<.001	Yes
H8a	MFA→PC	−0.112	1.970 (312)	.049	No
H8b	MLA→PC	0.105	1.379 (312)	.17	No
H9	PC→DI^f^	−0.012	0.340 (312)	.73	No
H10	TRU→DI	0.675	21.163 (312)	<.001	Yes
H11	TRU→SAT^g^	0.818	46.783 (312)	<.001	Yes
H12	TRU→CU^h^	0.190	4.874 (312)	<.001	Yes
H13	SAT→CU	0.736	19.964 (312)	<.001	Yes

^a^SP: social presence.

^b^TRU: trust.

^c^MFA: mindful anthropomorphism.

^d^MLA: mindless anthropomorphism.

^e^PC: privacy concerns.

^f^DI: intention to disclose information.

^g^SAT: satisfaction.

^h^CU: intention to continuously use.

## Discussion

### Principal Findings

The Chinese mHealth market has witnessed rapid expansion, and health care CAs are widely applied in various fields of web-based diagnosis and follow-up care. For our participants who already had use experience with mobile medical consultation services, we found that a majority of them (99/103) had interacted with intelligent guidance CAs before they met web-based physicians, which confirmed the necessity of building trust between them because such CAs are vital in collecting patients’ symptom information, redirecting patients to suitable physicians, and thus formulating the first impressions during mobile medical consultation services [3]. Nevertheless, it is rarely explored how humanlike attributes influence patients’ perceived anthropomorphism, social presence, trust building, and acceptance of intelligent guidance CAs. By conducting this research, first, we found that a high anthropomorphic level of agent appearance and verbal cues could improve the patients’ perceptions of mindful anthropomorphism and mindless anthropomorphism, as well as social presence. Second, we further found that mindless anthropomorphism and social presence significantly promoted patients’ trust in CAs, and mindful anthropomorphism and social presence helped decrease their privacy concerns. Third, this study revealed that trust was an important antecedent and determinant of patients’ acceptance of CAs, including their satisfaction, intention to disclose information, and intention to continuously use CAs. This study has theoretical and practical implications. Furthermore, the possible limitations of this study should also be considered.

### Effects of Anthropomorphic Cues on Perceived Anthropomorphism and Social Presence

To assess how anthropomorphic cues influence patients’ perceptions of intelligent guidance CAs, a 3×2 (agent appearance: high-level humanlike vs medium-level humanlike vs low-level humanlike; verbal cues: humanlike vs machine-like) within-subject factorial experiment was conducted.

As expected, this study found that participants perceived a higher level of mindful and mindless anthropomorphism with CAs with humanlike agent appearances and verbal cues (supporting H1 and H2), which revealed that people tend to attribute human characteristics to CAs in a mindful and mindless manner. In addition, the CAs designed with anthropomorphic cues could easily elicit a feeling of social presence (supporting H3), which implies the anthropomorphic cues’ capability of reminding patients of a humanlike personality on the interface [10,11,17,82]. To be specific, this study found that participants were likelier to perceive intelligent guidance CAs as natural, humanlike, conscious, lifelike (mindful anthropomorphism), likable, sociable, and friendly (mindless anthropomorphism) as well as likelier to perceive the CAs’ attitudes, imagine how they look, and feel their sense of human touch and warmth (social presence) when the agent appearances of CAs were designed with a humanlike cartoon avatar with some facial expressions, such as smiles, winks, and mouth movements, or when the CAs expressed some formulations of self-introduction, greetings, farewells, thanking, or tips and advice.

It is also interesting to note that there was a significant interaction between agent appearance and verbal cues when the agent appearance was at low and medium levels of anthropomorphism. The results suggest that if health care CAs are designed with low or medium anthropomorphic levels of agent appearance, the anthropomorphic level of verbal cues becomes more important in determining patients’ perceived anthropomorphism and social presence. In other words, designers and practitioners are advised to use humanlike verbal cues for health care CAs to facilitate the patients’ perceived anthropomorphism and social presence, if they do not have the chance to design an agent with a high anthropomorphic level of appearance.

Furthermore, this study is also helpful in addressing the argument about whether social responses to CAs occur in a mindful or mindless manner. Some of the results are consistent with previous research on general computers and websites [15,18], which suggests that participants usually unconsciously and mindlessly treat CAs in human terms, such as perceiving them as likable or personal. However, the results diverged from the aforementioned studies, indicating that individuals would mindfully refuse to perceive computers as humanlike entities directly [18]. This research suggests that participants may consciously and directly respond to intelligent guidance CAs as humanlike or lifelike entities, which confirms some of the previous research focusing on CAs [7,83]. There may be 2 reasons for this. First, the agent appearances and verbal cues used for the various anthropomorphic levels in this study were designed at an acceptable level to avoid overhumanization. Thus, the anthropomorphic cues may not have led to any uncanny feelings or overly high expectations from the participants [8,9]; therefore, the participants did not refuse to acknowledge the CAs as humanlike entities. Second, patients are usually quite sensitive and vulnerable in medical consultations because the diseases may pose high risks to their well-being. Participants may treat such CAs as different types of partners because they are eager to seek advice and help from them. In this vein, they may experience fewer difficulties and hesitations in mindfully applying humanlike attributes to intelligent guidance CAs rather than general computers or websites [7].

### How Perceived Anthropomorphism and Social Presence Facilitate the Trust Building Toward Health Care CAs

The second set of contributions in this study involves investigating what types of factors can facilitate or hinder patients’ trust building in human-agent interactions. The results revealed several meaningful insights into the participants’ trust and privacy concerns.

First, the participants’ perceptions of social presence were found to directly influence their trust in intelligent guidance CAs (supporting H4). The results agreed with the majority of previous studies supporting the finding that social presence can influence trust building in media communications [11,41,43]. Thus, the results reinforce the importance of delivering a higher level of social presence by assigning a humanlike personality or image to health care CAs to enable a sense of human touch and warmth during human-agent interactions. Second, unlike an earlier study reporting that social presence could induce a feeling of being watched and further induce privacy concerns [51], social presence in this study was found to significantly decrease the participants’ privacy concerns (supporting H7). This conforms to previous studies arguing that social presence can help shorten the human-agent distance and relieve patients’ anxiety and uncertainty in web-based environments [11,52]. We believe that the differences lie in the fact that the web-based medical environment is much more complex and sensitive than other web-based services such as e-commerce or customer service. When anthropomorphic cues provide a higher level of social presence, fewer privacy concerns are produced, and a higher level of trust toward CAs is developed. This may also explain why privacy concerns partially mediate the effects of social presence on trust [48,50]. Third, this study confirmed the point of view that privacy risk is one of the major hindrances to trust development in human-agent interaction [46,48,50,51]. Privacy concerns were reported to have a significant influence on the participants’ trust in intelligent guidance CAs (supporting H6). Thus, it is suggested that less concern about the loss of control over personal information and improper collection of access to such information could induce a higher level of trustworthiness in CAs.

Fourth, although earlier studies indicated the importance of anthropomorphism for building trust by strengthening the social bonds between patients and CAs, limited insights were captured from these studies [20,21,45]. To compensate for this, this study explored the effects of both mindful and mindless anthropomorphism on patients’ trust in intelligent guidance CAs. Mindless anthropomorphism, such as perceiving the CAs as “likable,” “sociable,” and “friendly,” was found to significantly facilitate the trust-building process but not impact privacy concerns (supporting H5b and rejecting H8b). This result was supported by previous studies emphasizing that the interpersonal trust models under the Computers Are Social Actors paradigm (ie, people automatically apply social heuristics while interacting with CAs) are still applicable to explain patients’ trust building with CAs [5,84]. Therefore, it is easier to develop trustworthiness between patients and intelligent guidance CAs when more personalities are mindlessly attributed to such CAs. Meanwhile, mindful anthropomorphism, such as perceiving the CAs as “natural,” “humanlike,” “conscious,” and “lifelike,” failed to directly benefit the trust-building process (rejecting H5a). The effect was found to be mainly mediated by the participants’ privacy concerns. Specifically, mindful anthropomorphism was found to influence participants’ privacy concerns marginally and negatively, which suggests that people have fewer privacy concerns when perceiving a higher level of mindful anthropomorphism (rejecting H8a). The results confirmed those of Ischen et al [50] and diverged from those of some previous studies [45,55,56]. A possible reason is that humanlike intelligent guidance CAs may lead to feelings of being helped rather than being watched, compared with customer service CAs. Therefore, an improved anthropomorphic level does not induce greater privacy concerns. In conclusion, this study complemented previous research by finding that mindfully perceiving intelligent guidance CAs as having a humanlike character is not enough to trigger a trust-building process and mindlessly attributing human personalities to intelligent guidance CAs is not necessary to relieve patients’ privacy concerns. In other words, in a highly vulnerable and complex web-based environment, such as that of mobile medical consultations, it is vital to attribute human personalities to intelligent guidance CAs in an unconscious and mindless manner to facilitate the trust-building process. Simultaneously, it is also helpful to mindfully attribute a human character to such CAs because it can help reduce patients’ privacy concerns and further facilitate trust building with the CAs.

### How Trust and Privacy Concerns Influence the Acceptance of Health Care CAs

The effects of trust and privacy concerns on participants’ acceptance behavior were also investigated in this study. Consistent with previous studies, trust played a critical role in predicting patients’ intention to disclose information [37-39,59], intention to continuously use the intelligent guidance CAs, and satisfaction [11,37,39,46,60,76] (supporting H10, H12, and H11). In particular, the effects of trust on the intention to disclose information and satisfaction were quite strong, but the effects of trust on the intention to continuously use CAs were very weak. It is worth noting that most of the effects of trust on the intention to continuously use CAs were mediated by the participants’ satisfaction. In other words, when intelligent guidance CAs are believed to be trustworthy and reliable as well as to keep commitments and meet participants’ expectations, patients are more likely to disclose information to these CAs and have a higher level of satisfaction with the CAs, which will further encourage continuous intelligent guidance CA use. However, unlike earlier studies reporting that privacy concerns could negatively influence participants’ intention to disclose information [57,58], the results of this study did not suggest a direct influence of privacy concerns on the participants’ intention to disclose information when using intelligent guidance CAs (rejecting H9). Instead, it was found that trust in CAs could fully mediate the causal relationship between privacy concerns and the intention to disclose information. In other words, although the amelioration of privacy concerns may not determine patients’ intention to disclose information in web-based triage services, it is quite helpful in improving their trust toward intelligent guidance CAs, thereby improving their willingness to disclose more information, such as personal details, disease records, or symptom information.

### Implications

This study has valuable theoretical and practical implications. Theoretically, the results of this study reinforce the importance of trust building in patients’ acceptance and adoption of health care CAs, which can contribute to extending the application of traditional technology acceptance and adoption models in the mHealth field. More precisely, the results revealed that trust in intelligent guidance CAs is one of the antecedents for enabling more information exchange and disclosure, improving patient satisfaction, and leading to the continuous adoption of such CAs. Meanwhile, trust building is a complex process that is influenced by patients’ mindless anthropomorphism, social presence, and privacy concerns. Practically, by identifying the effects of anthropomorphic cues on patients’ perceptions of intelligent guidance CAs, this study paved the way for designers and practitioners to attribute humanness to health care CAs. It has been reported that a humanlike agent appearance and verbal cues could significantly and positively influence patients’ perceptions of mindful and mindless anthropomorphism as well as social presence, which may further increase their trust toward intelligent guidance CAs while decreasing their privacy concerns. Therefore, designers and practitioners are advised to use simple facial expressions (ie, smiles, winks, and mouth movements) and humanlike verbal cues (ie, self-introduction, greetings, farewells, thanking, and tips and advice) to improve the CAs’ anthropomorphic level. However, in contrast with some previous studies [15,18], this study did not find any evidence that patients mindfully refuse to perceive CAs as humanlike entities, which may be because the humanlike cartoon avatar used in this study conformed to the stereotype of guiding nurses in the hospital. In addition, we did not study the associations between the CAs’ figures, stereotypes, and tasks. Accordingly, designers and practitioners are suggested to carefully implement humanlike cartoon avatars in other contexts of use.

Meanwhile, a notable ethical consequence that may be brought by “anthropomorphism by design” is the possible risk of “machine masquerading” [85]. As reported earlier, anthropomorphic CAs may invoke more trust than they truly deserve, and patients may be disclosing more information to CAs unconsciously [86]. Thus, it is vital to distinguish between honest and dishonest anthropomorphic designs [87]. Although the results of this study encourage designers to use anthropomorphic cues to facilitate patients’ trust in CAs and decrease their privacy concerns, the suggestions were proposed by assuming that CAs are designed with honest anthropomorphism. Nevertheless, designers are highly advised to carefully implement the suggestions of this study and be aware of both the intentional and unintentional varieties of dishonest anthropomorphism, for instance, giving too successful impressions of humanoid CAs or raising patients’ overblown expectations for such CAs [87,88]. A separate discussion is required in future studies to explore the relationships between dishonest anthropomorphism, trust, and privacy concerns.

### Limitations and Future Study

This study had some limitations. First, we conducted the experiment using a web-based distribution platform. Although 2 experts were invited to check the validation of the instrument items and wording before the experiment and an attention filter was designed to check any disqualified questionnaires, the external validity of the findings may be influenced to some extent. Second, the participants in this study were not diagnosed with any actual disease during the experiment. They were instructed to experience the hypothetical interaction scenarios and evaluate their perceived anthropomorphism, social presence, trust building, and acceptance behavior after watching the stimuli materials of the video vignettes. Future research could consider recruiting real patients experiencing possible treatments and risks from diseases and using physiological methods such as eye-tracking or electroencephalogram to measure the patients’ real-time reactions. Third, our sample was quite homogenous, comprising Chinese patients from resource-rich cities with a relatively high level of education and habitually adopting various types of information communication technologies for mobile medical consultation services. We did not consider nonusers of mobile medical consultation because they may face more hindrances when deciding whether they would accept the health care CAs or not, such as perceived risk, subjective norms, and facilitating conditions [5,89,90]. These hindrances may interact with the possible effects of health care CA design and induce unknown confounding results. We did not investigate the possible influences of patient characteristics such as age, sex, education level, income, cultural background, and technology experience. Future studies should further evaluate the possible effects of such patient-related factors with more heterogeneous samples. Furthermore, previous studies indicated that the perceived age and sex of CAs may influence users’ perception and acceptance of anthropomorphic technology [65,91,92]. Meanwhile, such effects vary among users of different sexes and ages as well as various service contexts [65,91]. Considering that the evidence is still inconclusive, we did not examine the effects of the age and sex stereotypes, elicited by the appearance design of intelligent guidance CAs, on patients’ perceived anthropomorphism, trust, and acceptance behavior in this study. Future studies are needed to further evaluate how age and sex stereotypes impact health care CAs’ trust and acceptance. In addition, trust building between patients and CAs tends to be a dynamic process. Trust may vary depending on the various stages of health care CA use and adoption. At the same time, this study did not use the actual use of health care CAs as the symbol of acceptance because it is quite difficult to measure participants’ actual use behavior in a web-based survey-based experiment. Instead, we measured the participants’ satisfaction, intention to disclose information, and intention to continuously use the intelligent guidance CAs, which have been widely used in studying technology acceptance in previous studies [11,37,89]. Follow-up studies are necessary to investigate the dynamic trust-building process and patients’ actual use of health care CAs over a long period. Finally, our findings focused on patients’ perceived anthropomorphism, social presence, trust building, and acceptance of health care CAs used for web-based triage services. It is possible that patients’ perceptions and expectations of health care CAs differ in other health care service contexts. The results should be carefully applied, depending on the service domain and health care tasks.

### Conclusions

This study provides valuable insights into patients’ perceived anthropomorphism, social presence, trust building, and acceptance of intelligent guidance CAs. The results suggest that the use of anthropomorphic cues, such as agent appearance and verbal cues, can significantly improve patients’ perceived anthropomorphism and social presence. Furthermore, mindless anthropomorphism and social presence further facilitate the process of trust development. Meanwhile, mindful anthropomorphism and social presence could help ameliorate privacy concerns. It is worth noting that trust in CAs is an important antecedent of patients’ acceptance of intelligent guidance CAs, which largely influences patients’ satisfaction, intention to disclose information, and intention to continuously use such CAs. Although privacy concerns did not have any direct effect on patients’ intention to disclose information, the explanatory relationship was found to be fully mediated by trust. To conclude, to facilitate patients’ acceptance and adoption of health care CAs, it is crucial to support the development of trust between patients and health care CAs. From the perspective of agent design, a suitably designed humanlike cartoon agent, which conforms to the stereotype associated with health care tasks, can help improve trustworthiness and reduce privacy concerns to some extent. Designers and practitioners are therefore suggested to attribute personal characteristics to CAs, such as simple facial expressions, and humanlike verbal cues, such as self-introductions, greetings, farewells, thanking, and tips and advice. In addition, considering the possible limitations of current studies, future studies should recruit more heterogeneous samples, use real-time physiological measures, investigate a dynamic process of trust building over a period, and evaluate the possible differences created by various health care contexts.

## Appendix

Multimedia Appendix 1 Examples of web-based triage services in mobile medical consultations.

Multimedia Appendix 2 An example of video vignettes developed for the experiment.

Multimedia Appendix 3 Survey on perceptions and acceptance of health care conversational agents.

Multimedia Appendix 4 The item-item correlation matrix of acceptance predictors.

Multimedia Appendix 5 Means, SDs, reliability, and validity of the partial least squares measurement model.

## Abbreviations

AVE: average variance extracted
CA: conversational agent
MAIN: Modality, Agency, Interactivity, and Navigability
mHealth: mobile health
PLS: partial least squares
RQ: research question
VAF: variance accounted for

## References

[1] Montenegro JL, da Costa CA, da Rosa Righi R (2019). Survey of conversational agents in health. Expert Syst Appl.

[2] Go E, Sundar SS (2019). Humanizing chatbots: the effects of visual, identity and conversational cues on humanness perceptions. Comput Hum Behav.

[3] Nadarzynski T, Miles O, Cowie A, Ridge D (2019). Acceptability of artificial intelligence (AI)-led chatbot services in healthcare: a mixed-methods study. Digit Health.

[4] Denecke K, Tschanz M, Dorner TL, May R (2019). Intelligent conversational agents in healthcare: hype or hope?. Stud Health Technol Inform.

[5] Seitz L, Bekmeier-Feuerhahn S, Gohil K (2022). Can we trust a chatbot like a physician? A qualitative study on understanding the emergence of trust toward diagnostic chatbots. Int J Hum Comput Stud.

[6] Neururer M, Schlögl S, Brinkschulte L, Groth A (2018). Perceptions on authenticity in chat bots. Multimodal Technol Interact.

[7] Araujo T (2018). Living up to the chatbot hype: the influence of anthropomorphic design cues and communicative agency framing on conversational agent and company perceptions. Comput Hum Behav.

[8] Chaves AP, Gerosa MA (2020). How should my chatbot interact? A survey on social characteristics in human–chatbot interaction design. Int J Hum Comput Interact.

[9] Ciechanowski L, Przegalinska A, Magnuski M, Gloor P (2019). In the shades of the uncanny valley: an experimental study of human–chatbot interaction. Future Gener Comput Syst.

[10] Zhang J, Li Q, Luximon Y (2021). Building trust in mobile medical consultations: the roles of privacy concerns, personality traits, and social cues. Human Aspects of IT for the Aged Population. Technology Design and Acceptance.

[11] Zhang J, Luximon Y, Li Q (2022). Seeking medical advice in mobile applications: how social cue design and privacy concerns influence trust and behavioral intention in impersonal patient–physician interactions. Comput Hum Behav.

[12] Gottliebsen K, Petersson G (2020). Limited evidence of benefits of patient operated intelligent primary care triage tools: findings of a literature review. BMJ Health Care Inform.

[13] Comendador BE, Francisco BM, Medenilla JS, Nacion SM, Serac TB (2015). Pharmabot: a pediatric generic medicine consultant chatbot. J Open Autom Control Eng.

[14] Hoermann S, McCabe KL, Milne DN, Calvo RA (2017). Application of synchronous text-based dialogue systems in mental health interventions: systematic review. J Med Internet Res.

[15] Nass C, Moon Y (2000). Machines and mindlessness: social responses to computers. J Social Isssues.

[16] Esfahani MS, Reynolds N, Ashleigh M (2020). Mindful and mindless anthropomorphism: how to facilitate consumer comprehension towards new products. Int J Innov Technol Manag.

[17] Feine J, Gnewuch U, Morana S, Maedche A (2019). A taxonomy of social cues for conversational agents. Int J Hum Comput Stud.

[18] Kim Y, Sundar SS (2012). Anthropomorphism of computers: is it mindful or mindless?. Comput Hum Behav.

[19] Shyam SS, Metzger MJ, Flanagin AJ (2008). The MAIN model: a heuristic approach to understanding technology effects on credibility. The John D. and Catherine T. MacArthur Foundation Series on Digital Media and Learning.

[20] Beun RJ, de Vos E, Witteman C, Rist T, Aylett RS, Ballin D, Rickel J (2003). Embodied conversational agents: effects on memory performance and anthropomorphisation. Intelligent Virtual Agents.

[21] Kang H, Kim KJ (2020). Feeling connected to smart objects? A moderated mediation model of locus of agency, anthropomorphism, and sense of connectedness. Int J Hum Comput Stud.

[22] Posard MN, Rinderknecht RG (2015). Do people like working with computers more than human beings?. Comput Hum Behav.

[23] Antaki C, Peräkylä A, Antaki C, Vehviläinen S, Leudar I (2008). Formulations in psychotherapy. Conversation Analysis and Psychotherapy.

[24] Cassell J (2000). Embodied conversational interface agents. Commun ACM.

[25] Ryokai K, Boulanger C, Cassell J (2003). Virtual peers as partners in storytelling and literacy learning. J Comput Assist Learn.

[26] Collier G (1985). Emotional Expression.

[27] Bartneck C, Kulić D, Croft E, Zoghbi S (2008). Measurement instruments for the anthropomorphism, animacy, likeability, perceived intelligence, and perceived safety of robots. Int J of Soc Robotics.

[28] Gong L, Nass C (2007). When a talking-face computer agent is half-human and half-humanoid: human identity and consistency preference. Hum Comm Res.

[29] Short J, Williams E, Christie B (1976). The Social Psychology of Telecommunications.

[30] Choi S (2016). The flipside of ubiquitous connectivity enabled by smartphone-based social networking service: social presence and privacy concern. Comput Hum Behav.

[31] Biocca F, Harms C, Burgoon JK (2003). Toward a more robust theory and measure of social presence: review and suggested criteria. Presence Teleoperators Virtual Environ.

[32] Skalski P, Brahnam S, Jain LC (2010). The role of presence in healthcare technology applications. Advanced Computational Intelligence Paradigms in Healthcare 5.

[33] Sutcliffe AG, Wang D, Dunbar RI (2015). Modelling the role of trust in social relationships. ACM Trans Internet Technol.

[34] Gefen D, Benbasat I, Pavlou P (2014). A research agenda for trust in online environments. J Manag Inf Syst.

[35] Rempel JK, Holmes JG, Zanna MP (1985). Trust in close relationships. J Pers Soc Psychol.

[36] Pearson SD, Raeke LH (2000). Patients' trust in physicians: many theories, few measures, and little data. J Gen Intern Med.

[37] Li Q (2020). Healthcare at your fingertips: the acceptance and adoption of mobile medical treatment services among Chinese users. Int J Environ Res Public Health.

[38] Bao Y, Hoque R, Wang S (2017). Investigating the determinants of Chinese adult children's intention to use online health information for their aged parents. Int J Med Inform.

[39] Deng Z, Liu S, Hinz O (2015). The health information seeking and usage behavior intention of Chinese consumers through mobile phones. Inf Technol People.

[40] Hillen MA, Postma R-M, Verdam MG, Smets EM (2017). Development and validation of an abbreviated version of the Trust in Oncologist Scale-the Trust in Oncologist Scale-short form (TiOS-SF). Support Care Cancer.

[41] Erfani SS, Abedin B, Blount Y (2016). The effect of social network site use on the psychological well-being of cancer patients. J Assoc Inf Sci Technol.

[42] Hawkins RP, Han J-Y, Pingree S, Shaw BR, Baker TB, Roberts LJ (2010). Interactivity and presence of three eHealth interventions. Comput Hum Behav.

[43] Lazard AJ, Brennen JS, Troutman Adams E, Love B (2020). Cues for increasing social presence for mobile health app adoption. J Health Commun.

[44] Walther JB, Pingree S, Hawkins RP, Buller DB (2005). Attributes of interactive online health information systems. J Med Internet Res.

[45] Ha QA, Chen JV, Uy HU, Capistrano EP (2020). Exploring the privacy concerns in using intelligent virtual assistants under perspectives of information sensitivity and anthropomorphism. Int J Hum Comput Interact.

[46] Yang M, Jiang J, Kiang M, Yuan F (2022). Re-examining the impact of multidimensional trust on patients' online medical consultation service continuance decision. Inf Syst Front.

[47] Fox G, James TL (2020). Toward an understanding of the antecedents to health information privacy concern: a mixed methods study. Inf Syst Front.

[48] Saffarizadeh K, Boodraj M, Alashoor T (2017). Conversational assistants: investigating privacy concerns, trust, and self-disclosure. Proceedings of the 38th International Conference on Information Systems.

[49] Smith HJ, Milberg SJ, Burke SJ (1996). Information privacy: measuring individuals' concerns about organizational practices. MIS Q.

[50] Ischen C, Araujo T, Voorveld H, van NG, Smit E (2020). Privacy concerns in chatbot interactions. Chatbot Research and Design.

[51] Sohn S (2019). Can conversational user interfaces be harmful? The undesirable effects on privacy concern. Proceedings of the 40th International Conference on Information Systems (ICIS).

[52] Hong W, Chan FK, Thong JY (2019). Drivers and inhibitors of internet privacy concern: a multidimensional development theory perspective. J Bus Ethics.

[53] Choi YK, Miracle GE, Biocca F (2001). The effects of anthropomorphic agents on advertising effectiveness and the mediating role of presence. J Interact Advert.

[54] Kim S, McGill AL (2011). Gaming with Mr. Slot or gaming the slot machine? Power, anthropomorphism, and risk perception. J Consum Res.

[55] Petronio S (2002). Boundaries of Privacy Dialectics of Disclosure.

[56] Xie Y, Chen K, Guo X (2020). Online anthropomorphism and consumers’ privacy concern: moderating roles of need for interaction and social exclusion. J Retail Consum Serv.

[57] Taddei S, Contena B (2013). Privacy, trust and control: which relationships with online self-disclosure?. Comput Hum Behav.

[58] Zhang X, Liu S, Chen X, Wang L, Gao B, Zhu Q (2018). Health information privacy concerns, antecedents, and information disclosure intention in online health communities. Inf Manag.

[59] Hall MA, Camacho F, Dugan E, Balkrishnan R (2002). Trust in the medical profession: conceptual and measurement issues. Health Serv Res.

[60] Bansal G, Zahedi FM, Gefen D (2010). The impact of personal dispositions on information sensitivity, privacy concern and trust in disclosing health information online. Decis Support Syst.

[61] Davis FD, Bagozzi RP, Warshaw PR (1989). User acceptance of computer technology: a comparison of two theoretical models. Manag Sci.

[62] Lehmann S, Ruf E, Misoch S, Ziefle M, Guldemond N, Maciaszek LA (2021). Emotions and attitudes of older adults toward robots of different appearances and in different situations. Information and Communication Technologies for Ageing Well and e-Health.

[63] Chita-Tegmark M, Ackerman JM, Scheutz M (2019). Effects of assistive robot behavior on impressions of patient psychological attributes: vignette-based human-robot interaction study. J Med Internet Res.

[64] Biswas M, Romeo M, Cangelosi A, Jones RB (2019). Are older people any different from younger people in the way they want to interact with robots? Scenario based survey. J Multimodal User Interfaces.

[65] Pak R, McLaughlin AC, Bass B (2014). A multi-level analysis of the effects of age and gender stereotypes on trust in anthropomorphic technology by younger and older adults. Ergonomics.

[66] Bavafa H, Hitt LM, Terwiesch C (2018). The impact of e-visits on visit frequencies and patient health: evidence from primary care. Manage Sci.

[67] Guo X, Han X, Zhang X, Dang Y, Chen C (2015). Investigating m-health acceptance from a protection motivation theory perspective: gender and age differences. Telemed J E Health.

[68] Sun Y, Wang N, Guo X, Peng JZ (2013). Understanding the acceptance of mobile health services: a comparison and integration of alternative models. J Electron Commer Res.

[69] Chang M-Y, Pang C, Tarn JM, Liu T-S, Yen DC (2015). Exploring user acceptance of an e-hospital service: an empirical study in Taiwan. Comput Stand Interfaces.

[70] Guo X, Zhang X, Sun Y (2016). The privacy–personalization paradox in mHealth services acceptance of different age groups. Electron Commer Res Appl.

[71] Lu B, Fan W, Zhou M (2016). Social presence, trust, and social commerce purchase intention: an empirical research. Comput Hum Behav.

[72] Akter S, D'Ambra J, Ray P (2010). Trustworthiness in mHealth information services: an assessment of a hierarchical model with mediating and moderating effects using partial least squares (PLS). J Am Soc Inf Sci.

[73] McKnight DH, Choudhury V, Kacmar C (2002). Developing and validating trust measures for e-commerce: an integrative typology. Inform Syst Res.

[74] Teo TS, Liu J (2007). Consumer trust in e-commerce in the United States, Singapore and China. Omega.

[75] Featherman MS, Pavlou PA (2003). Predicting e-services adoption: a perceived risk facets perspective. Int J Hum Comput Stud.

[76] Chen Y, Yang L, Zhang M, Yang J (2018). Central or peripheral? Cognition elaboration cues' effect on users' continuance intention of mobile health applications in the developing markets. Int J Med Inform.

[77] Sharma S, Crossler RE (2014). Disclosing too much? Situational factors affecting information disclosure in social commerce environment. Electron Commer Res Appl.

[78] Mishra P, Pandey CM, Singh U, Gupta A, Sahu C, Keshri A (2019). Descriptive statistics and normality tests for statistical data. Ann Card Anaesth.

[79] Hair JF, Ringle CM, Sarstedt M (2014). PLS-SEM: indeed a silver bullet. J Mark Theory Pract.

[80] Fornell C, Larcker DF (2018). Evaluating structural equation models with unobservable variables and measurement error. J Market Res.

[81] Chin WW (1998). Commentary: issues and opinion on structural equation modeling. MIS Q.

[82] Sah YJ, Peng W (2015). Effects of visual and linguistic anthropomorphic cues on social perception, self-awareness, and information disclosure in a health website. Comput Hum Behav.

[83] Warwick K, Shah H (2015). The importance of a human viewpoint on computer natural language capabilities: a Turing test perspective. AI Soc.

[84] Wang W, Benbasat I (2016). Empirical assessment of alternative designs for enhancing different types of trusting beliefs in online recommendation agents. J Manag Inform Syst.

[85] Miller K, Wolf MJ, Grodzinsky F (2014). Behind the mask: machine morality. J Exp Theor Artif Intell.

[86] Salles A, Evers K, Farisco M (2020). Anthropomorphism in AI. AJOB Neurosci.

[87] Kaminski ME, Rueben M, Smart WD, Grimm C (2017). Averting robot eyes. Maryland Law Rev.

[88] Leong B, Selinger E (2019). Robot eyes wide shut: understanding dishonest anthropomorphism. Proceedings of the Conference on Fairness, Accountability, and Transparency.

[89] Wang H, Tao D, Yu N, Qu X (2020). Understanding consumer acceptance of healthcare wearable devices: an integrated model of UTAUT and TTF. Int J Med Inform.

[90] Tao D, Wang T, Wang T, Zhang T, Zhang X, Qu X (2020). A systematic review and meta-analysis of user acceptance of consumer-oriented health information technologies. Comput Hum Behav.

[91] Seo S (2022). When Female (Male) Robot Is Talking To Me: effect of service robots’ gender and anthropomorphism on customer satisfaction. Int J Hosp Manag.

[92] Roesler E, Naendrup-Poell L, Manzey D, Onnasch L (2022). Why context matters: the influence of application domain on preferred degree of anthropomorphism and gender attribution in human–robot interaction. Int J of Soc Robotics.

