# Supplementary material for: The Influence of Anthropomorphic Cues on Patients’ Perceived Anthropomorphism, Social Presence, Trust Building, and Acceptance of Health Care Conversational Agents: Within-Subject Web-Based Experiment
Source: J Med Internet Res. 2023 Aug 10;25:e44479. doi: 10.2196/44479 (PMC10450539; doi:10.2196/44479)

**Multimedia Appendix 1**

Examples of online triage services in MMCs: (a) Chunyu Doctor, (b) Ping An Health, and (c) Ali Health.


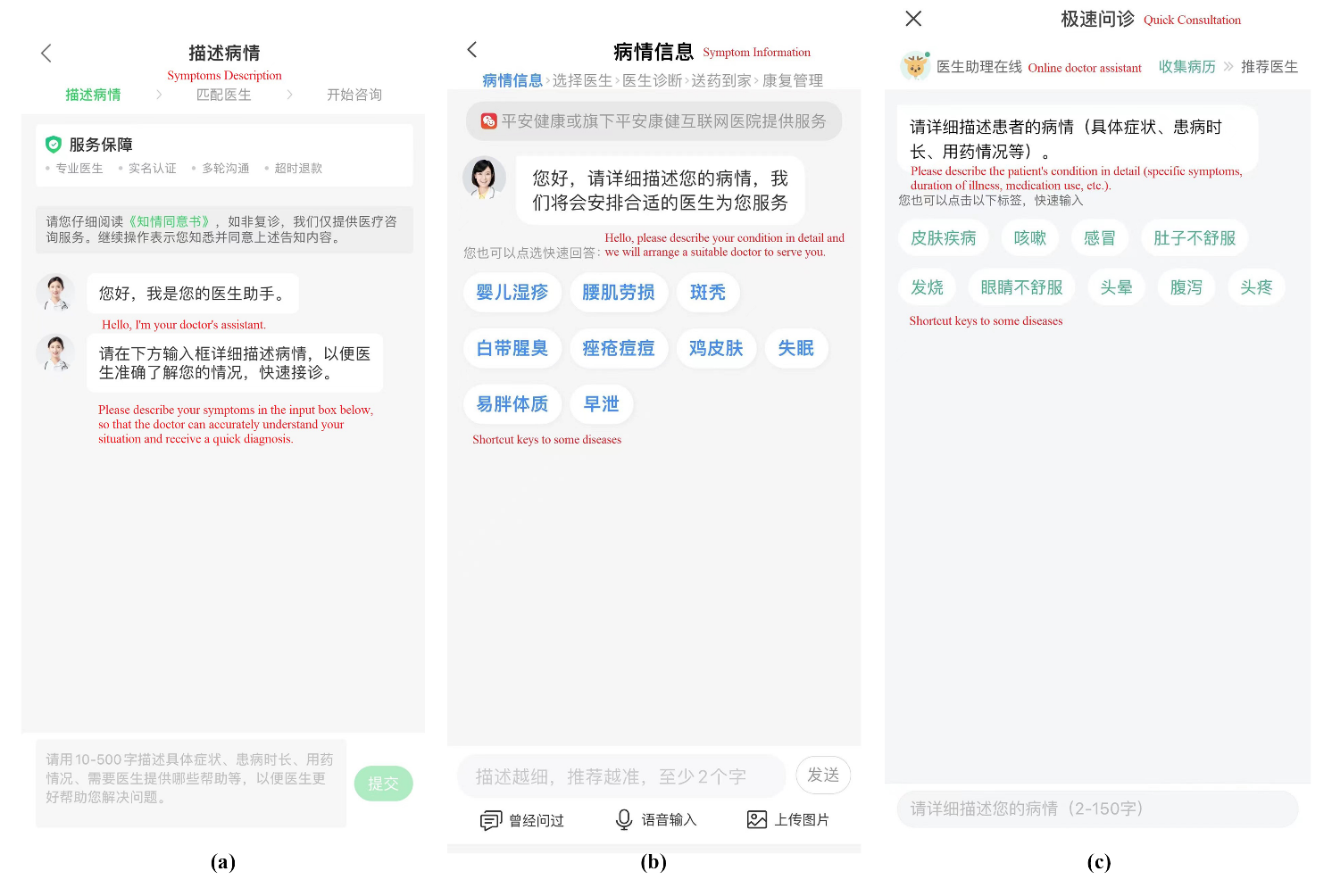

Supplement: Multimedia Appendix 1 [file jmir_v25i1e44479_app1.docx]
