# Supplementary material for: The Influence of Anthropomorphic Cues on Patients’ Perceived Anthropomorphism, Social Presence, Trust Building, and Acceptance of Health Care Conversational Agents: Within-Subject Web-Based Experiment
Source: J Med Internet Res. 2023 Aug 10;25:e44479. doi: 10.2196/44479 (PMC10450539; doi:10.2196/44479)
Supplement: Multimedia Appendix 3 [file jmir_v25i1e44479_app3.docx]

**Survey on Perceptions and Acceptance of Healthcare Conversational Agents**

**Part Ⅰ: Usage Experience**

1. Have you ever used the mobile medical treatment services (hereinafter referred to as MMC services) provided by mHealth apps for an online consultation?

For example, by sending text-, pictures-, voice-based messages, or through voice and video chat, you have consulted with online doctors through mHealth apps. The keywords of such services and functions usually include “ask the doctor”, “rapid inquiry”, “online diagnosis”, “professional inquiry”, and “emergency treatment”.

Yes  No

1. Please choose the mHealth app where you received the MMC services：

Weixin Smart Hospital

Ali Health

Jingdong Health

Ping An Good Doctor

Chunyu Doctor

Good Doctor online

Wedoctor

Health160

Miaoshou Doctor

Yihe Health

Daxiang Doctor

Zhuozheng Health

Others: ________

1. Have you ever used the online patient triage in the mobile medical consultations?

Yes  No

**Part Ⅱ Demographic Information**

1. Gender：  Male  Female
2. Age：________
3. The city where you currently live：________
4. Level of education：

Primary school  Middle school  High school  Undergraduate  Postgraduate and above

**Part Ⅲ Usage Behavior**

The following questions ask about your usage behavior with the MMC services. Please choose the option that best suits your personal situation:

1. Please choose the mHealth app where you received the MMC services：

Weixin Smart Hospital

Ali Health

Jingdong Health

Ping An Good Doctor

Chunyu Doctor

Good Doctor online

Wedoctor

Health160

Miaoshou Doctor

Yihe Health

Daxiang Doctor

Zhuozheng Health

Others: ________

1. How long have you been using the MMC services?

less than one year

one to two years

two to three years

more than three years

1. How many times have you used MMC services：

1-2 times

3-5 times

More than 5 times

I’m not sure

1. When using MMC services, which departments have you visited：

Internal medicine

Surgery

E.N.T

Dermatology

Obstetrics and gynecology

Psychology

Ophthalmology

Orthopedics

Pediatrics

Stomatology

Others:________

1. When you are using the above mHealth apps to consult with doctors online, which of the following communication methods did you choose?

Text messaging

Photo messaging

voice messaging

Calling

Voice chatting

Video chatting

Group chatting

Others:________

**Part Ⅳ Intelligent Guidance CA Experience and Evaluations**

Next, we will provide you with 6 kinds of intelligent guidance CAs in a video. Please browse these videos, and assume that you are a real user of the consultation service system. Afterwards, please rate each intelligent guidance CA based on your subjective experience. There is no right or wrong answer, please choose the option that best suits your actual situation.

1. Please indicate the No. of the video that you just finished watching.

1

2

3

4

5

6

1. Please evaluate your perceived anthropomorphism of the intelligent guidance CA.

| **Mindful Anthropomorphism:** How do you think of the intelligent guidance CA? | | | |  |
| --- | --- | --- | --- | --- |
| Fake | \|  \| \| --- \| | | Natural |  |
| Machine-like |  | | Human-like |  |
| Unconscious |  | | Conscious |  |
| Artificial |  | | Lifelike |  |
| **Mindless Anthropomorphism:** How do you think of the intelligent guidance CA? | | | | |
| I think the intelligent guidance CA is likeable. | | \| Strongly disagree strongly agree \| \| --- \| | | |
| I think the intelligent guidance CA is sociable. | | not agree at all strongly agree | | |
| I think the intelligent guidance CA is friendly. | | not agree at all strongly agree | | |

1. Please evaluate your perception of social presence regarding intelligent guidance CA.

| **Social presence:** please choose the option that best suits your actual situation. | |
| --- | --- |
| I can make sense of the attitude of the intelligent guidance CA by interacting with it. | \| Strongly disagree strongly agree \| \| --- \| |
| I can imagine how the intelligent guidance CA may look by interacting with it. | \| Strongly disagree strongly agree \| \| --- \| |
| There is a sense of human touch to communicate with the intelligent guidance CA. | \| Strongly disagree strongly agree \| \| --- \| |
| It is warm when communicating with the intelligent guidance CA. | \| Strongly disagree strongly agree \| \| --- \| |

1. The following questions ask about your perceptions regarding the intelligent guidance CA acceptance and adoption. Please indicate your perceptions from 1 (strongly disagree) to 7 (strongly agree).

| **Trust** | |
| --- | --- |
| I think the intelligent guidance CA is trustworthy. | \| Strongly disagree strongly agree \| \| --- \| |
| I think the intelligent guidance CA can provide reliable information. | \| Strongly disagree strongly agree \| \| --- \| |
| I think the intelligent guidance CA can keep promises and commitments | \| Strongly disagree strongly agree \| \| --- \| |
| I think the intelligent guidance CA can meet my expectations. | \| Strongly disagree strongly agree \| \| --- \| |
| **Privacy Concern** | |
| I think the intelligent guidance CA make me lose control over my privacy of information. | \| Strongly disagree strongly agree \| \| --- \| |
| I think the intelligent guidance CA may cause privacy problems. | \| Strongly disagree strongly agree \| \| --- \| |
| I think the intelligent guidance CA can lead to a loss of privacy because my personal information could be used without my knowledge. | \| Strongly disagree strongly agree \| \| --- \| |
| I think the intelligent guidance CA can make others take control of my information | \| Strongly disagree strongly agree \| \| --- \| |
| **Satisfaction** | |
| I think it’s a good idea to use the intelligent guidance CA. | \| Strongly disagree strongly agree \| \| --- \| |
| I think it is wise to use the intelligent guidance CA | \| Strongly disagree strongly agree \| \| --- \| |
| I like the idea of making use of the intelligent guidance CA. | \| Strongly disagree strongly agree \| \| --- \| |
| **Intention to disclose information** | |
| I am likely to provide my personal information and health information when using the intelligent guidance CA. | \| Strongly disagree strongly agree \| \| --- \| |
| I plan to provide my personal information and health information when using the intelligent guidance CA. | \| Strongly disagree strongly agree \| \| --- \| |
| I intend to provide my personal information and health information when using the intelligent guidance CA. | \| Strongly disagree strongly agree \| \| --- \| |
| **Intention to continuously use** | |
| I intend to use the intelligent guidance CA in the future. | \| Strongly disagree strongly agree \| \| --- \| |
| I believe I will use the intelligent guidance CA in the future. | \| Strongly disagree strongly agree \| \| --- \| |
| I plan to use the intelligent guidance CA in the future. | \| Strongly disagree strongly agree \| \| --- \| |

The survey is completed. Thank you for your participation!
